# Development of a novel certified reference material for the determination of polycyclic aromatic hydrocarbons (PAHs) in whey protein powder

**DOI:** 10.1007/s00216-023-04863-9

**Published:** 2023-07-28

**Authors:** Simon Lobsiger, Lena Märki, Silvia Mallia, Gisela Umbricht, Hanspeter Sprecher, Kathrin Breitruck, Markus Obkircher

**Affiliations:** 1 Federal Institute of Metrology METAS, Lindenweg 50, 3003 Bern-Wabern, Switzerland; 2 Sigma-Aldrich Production GmbH (a Subsidiary of Merck KGaA, Darmstadt, Germany), Industriestrasse 25, 9471 Buchs, Switzerland; 3 Sigma-Aldrich Chemicals Pvt Ltd (a subsidiary of Merck KGaA, Darmstadt, Germany), Plot No-12, Bommasandra Jigani Link Rd, Bengaluru, Karnataka 560099 India

**Keywords:** Certified reference material, PAHs, Whey protein powder, Food safety, GC–MS/MS, Solvent extraction efficiency

## Abstract

Matrix-based certified reference materials (CRMs) play a central role in the analysis of food contaminants for ensuring measurement accuracy and comparability, as they exhibit the same matrix effects during sample preparation and measurement as the food sample under investigation. However, the availability of such CRMs is still limited. This is also true for matrix CRMs containing polycyclic aromatic hydrocarbons (PAHs), for which maximum levels in food are set in the EU by the Commission Regulation (EU) 2023/915 and in Switzerland by the regulation SR 817.022.15. Therefore, a whey protein powder–based certified reference material (WP-CBR001) was developed according to the requirements of ISO 17034 and the recommendations of ISO Guide 35:2017 containing the four PAHs benz[*a*]anthracene (BaA), benzo[*a*]pyrene (BaP), benzo[*b*]fluoranthene (BbF), and chrysene (Chr). We show that the choice of solvent is of crucial importance to extract the PAHs completely from this matrix. Only polar and protic solvents such as methanol or water provided access for complete extraction of the PAHs. In contrast, nonpolar and polar aprotic solvents, such as n-hexane or ethyl acetate, exhibited only very low extraction efficiencies below 20%. The certified mass fractions and expanded uncertainties (*k* = 2) were (3.17 ± 0.32) µg/kg BaA, (4.18 ± 0.48) µg/kg BaP, (4.73 ± 0.49) µg/kg BbF, and (2.85 ± 0.33) µg/kg Chr. These values were verified by an interlaboratory comparison study and by the gravimetric mass fractions obtained from production data.

**Graphical abstract:**

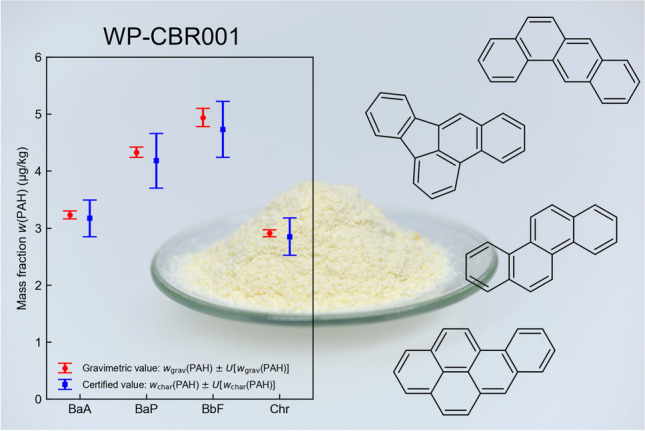

**Supplementary information:**

The online version contains supplementary material available at 10.1007/s00216-023-04863-9.

## Introduction

In a recent editorial in the bulletin of the World Health Organization (WHO), Choudhury et al. [1] call for a better study of the impact of chemicals on the foodborne burden of disease. Among other things, they note that better surveillance data are needed to capture the incidence of dietary exposure to chemical contaminants. In order to support these data, accurate and traceable measurements of chemical contaminants in foods are essential.

The analysis of chemical contaminants in foods generally requires a complex and matrix-dependent sample preparation and measurement procedure, including extraction, clean-up, and measurement. Certified reference materials (CRMs) play a key role in the development, validation, and performance assessment of analytical methods and the establishment of metrological traceability to reference values obtained by agreed realizations of the SI units, which is also a requirement for laboratories with ISO/IEC 17025 [2] accreditation. However, the current availability of CRMs in food matrices is small compared to a large number of chemical contaminants, matrices, and concentration ranges found in real foods. Therefore, there is a great need for the production and supply of new CRMs, which ultimately contribute to food safety.

The presence of polycyclic aromatic hydrocarbons (PAHs) in food represents an important hazard to human health. It has been shown that for non-smokers, diet is the major route of human exposure to PAHs [3, 4]. PAHs are a large class of organic compounds that consist of hydrocarbons with at least two fused aromatic ring systems. PAHs can enter into food products through different ways. Food, mainly cereals and meat, can be contaminated by PAHs through their presence in the environment [3]. PAHs are in fact ubiquitous environmental pollutants and occur in the air, soils, and water, mainly as a result of incomplete combustion of organic matter [5]. As such, vehicle motors, petroleum refineries, and power plants constitute the main anthropogenic sources of PAHs [6–8]. Furthermore, PAHs can form in food during industrial processing or domestic food preparation such as drying, heating, and grilling [9]. A third route for PAH contamination is through packaging processes and materials, e.g., through contact with mineral oils [10, 11]. Their low solubility in water and lipophilic characteristic allow PAHs to easily accumulate in food products.

**Fig. 1:**
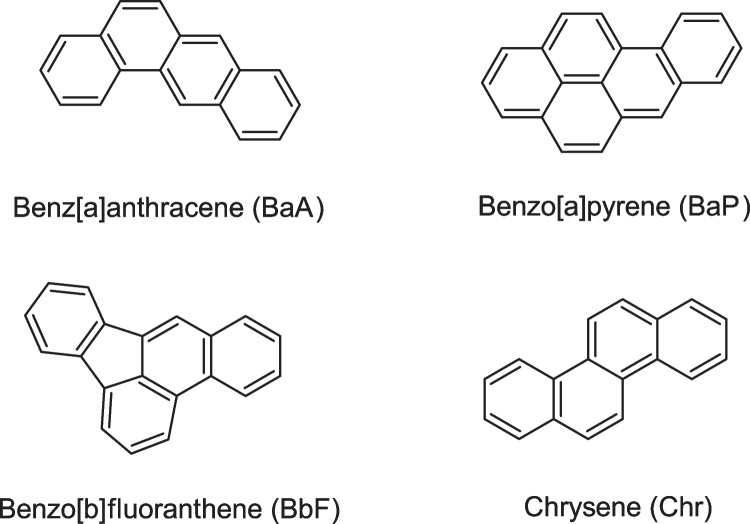
Polycyclic aromatic hydrocarbons (PAHs) in the certified reference material WP-CBR001

Among other studies, the Joint FAO/WHO Expert Committee on Food Additives stated that at least 13 PAHs are genotoxic and carcinogenic [12]. Benzo[a]pyrene (BaP) (see Fig. 1), the most studied PAH, has been classified by the International Agency for Research on Cancer (IARC) as “carcinogenic to humans,” while several other PAHs are classified as “probably carcinogenic to humans” [13, 14]. The mass fractions of PAHs in foods are therefore regulated in the European Union (EU) by the Commission Regulation (EU) 2023/915 [15] and in Switzerland by the regulation SR 817.022.15 on the maximum levels for contaminants [16]. The regulations exclusively concern benzo[*a*]pyrene (BaP) and the sum of benz[*a*]anthracene (BaA), benzo[*a*]pyrene (BaP), benzo[*b*]fluoranthene (BbF), and chrysene (Chr) in different food products.

Although whey protein is not explicitly listed as a foodstuff in these regulations, it can be used for mimicking high protein–containing matrices. Whey protein powders are produced from whey, which is a liquid by-product of cheese manufacturing and is therefore available in large quantities in the dairy industry. Due to its high content of essential elements and amino acids, whey protein powder has a high nutritional value and is one of the most commonly used food additives worldwide.

As such, whey protein has positive effects on muscle development, on the immune system, and on body weight loss, for example [17]. It is popular among athletes as it enables rapid recovery and muscle building after exercise [18, 19], and it is a high-protein source in enteral nutrition used in patient care [20, 21]. Additionally, whey protein powder has useful physico-chemical properties for food production and is often used in the food industry as an emulsifying agent or stabilizer [22]. Hence, whey protein can be found in many different food products, such as sports nutrition, protein drinks, baby food, infant formula, health-boosting food supplements, cakes, sausages, and cheese. However, as with many other food additives and supplements, whey protein may also be susceptible to contamination of PAHs through environmental pollution, production processes, or packaging materials.

Here, we present the development of WP-CBR001, a CRM for the determination of the four PAHs BaA, BaP, BbF, and Chr in a high-protein (approx. 0.8 g/g based on dry matter) matrix of whey protein powder.

## Materials and methods

### Design of the CRM development

The development of WP-CBR001 was performed as a joint project between the Federal Institute of Metrology (METAS), Sigma-Aldrich Production GmbH (a subsidiary of Merck KGaA, Darmstadt, Germany, hereafter referred to as Merck), and Hochdorf Swiss Nutrition Solutions AG (hereafter referred to as Hochdorf) and consisted of the following main steps: production planning, material production, analytical method, homogeneity and stability studies, characterization, assignment of property values and their verification by gravimetric mass fractions, and an interlaboratory comparison (ILC) study.

The target analytes in the CRM were defined as the PAHs BaA, BaP, BbF, and Chr and the toxic elements As, Cd, Hg, and Pb. Only the PAH part of the CRM development is reported here. The toxic element part is described elsewhere. BaA, BaP, BbF, and Chr were selected because maximum levels are set exclusively for these components by the Commission Regulation (EU) 2023/915 [15] in the EU and in Switzerland by the regulation SR 817.022.15 on the maximum levels for contaminants [16] in various foods.

Because industrially manufactured whey protein powder was shown to be free of the target PAHs (< 0.05 µg/kg), liquid whey was contaminated before further processing (spray drying) to the final product. The production process was chosen to be almost identical to that used in industry, in order to establish a CRM that mimics the sample preparation behavior of real whey protein powder. Since the mass fractions of PAHs in whey protein powders are not explicitly regulated in the EU and in Switzerland, the target mass fraction for each of the PAHs was chosen to be in the range of 1 µg/kg to 10 µg/kg, which corresponds to the regulated maximum levels of BaP (1 µg/kg to 10 µg/kg) and of the sum of BaA, BaP, BbF, and Chr (1 µg/kg and 50 µg/kg) in various foods.

Certification of WP-CBR001 was planned based on an in-house study in accordance with ISO 17034 [23] and ISO Guide 35:2017 [24]. In order to support the in-house certification strategy, the certified values were verified by the gravimetric mass fractions obtained from the production of WP-CBR001 and by an ILC study. This novel CRM allows the evaluation of the performance and the validation of analytical methods for the determination of PAHs in high-protein matrices.

### Material production

The whey raw material, a retentate from membrane filtration with approx. 0.32 g/g of dry matter, was supplied by Hochdorf from one of their conventional production streams. A quantity of 5 kg of this whey raw material was transferred to Merck where it was contaminated with the four PAHs BaA, BaP, BbF, and Chr using a spike solution in acetonitrile. The spike solution was prepared from a PAHs stock solution that was in turn prepared by dissolving CRMs of BaA (Sigma-Aldrich, Buchs, Switzerland, Supelco® 75451), BaP (Sigma-Aldrich, Buchs, Switzerland, Supelco® 51﻿968), BbF (Sigma-Aldrich, Buchs, Switzerland, Supelco® 30958), and Chr (Sigma-Aldrich, Buchs, Switzerland, Supelco® 94035) in acetonitrile. After spiking, the contaminated whey was stirred for 15 min at room temperature with a cup stirrer before it was transferred back to Hochdorf. There, the contaminated whey was mixed with another 85 kg portion of uncontaminated whey raw material, resulting in a total of 90 kg of contaminated whey. This mixture was then spray-dried using an industrial pilot plant yielding 23 kg of whey protein powder with approx. 0.77 g/g of protein (0.81 g/g of protein in dry matter), 0.10 g/g of carbohydrates, 0.06 g/g of fat, and 0.05 g/g of water. These are typical values of the main constituents of a commercial whey protein powder produced under similar conditions as WP-CBR001. After sieving, the final bulk product was transferred to Merck where it was filled into pre-cleaned amber glass bottles in 30 g portions. The total number of bottles (units) produced was *N*_prod_ = 678. The bottles were numbered according to the order of bottling. The bottles were stored at − 20 °C directly after filling. For the homogeneity and stability studies, selected bottles were stored separately at the appropriate storage temperatures. The production steps are schematically shown in Fig. S1 in the Supplementary Information.

### Analytical method

Mass fractions of the target analytes were obtained by gas chromatography (GC) linear regression isotope dilution mass spectrometry (IDMS). For the preparation of the calibration (reference) blends, the standard reference material SRM 1647f (NIST, Gaithersburg, USA) was used containing all relevant native PAHs dissolved in acetonitrile. The isotopically enriched spike (deuterated PAHs) was prepared from the “Deuterated IS All-in-one 16 EPA Priority PAHs” mix (Chiron AS, Trondheim, Norway, S-4513-K-T) dissolved in toluene. For the determination of the recovery of the spike (deuterated PAHs), an injection standard made from 9-fluorobenzo[*k*]fluoranthene (FBkF) (Chiron AS, Trondheim, Norway, 1322.20–100-T) dissolved in toluene was used. All preparation steps were performed gravimetrically. All masses were obtained by air buoyancy correction of the corresponding weights.

#### Sample blends

The preparation of the sample blends can mainly be divided into an extraction and a clean-up step. For the extraction of the test material, the accelerated solvent extraction (ASE) system EDGE (CEM Corporation) was used. A sample amount of 3 g was weighed into an EDGE Q-cup equipped with a C9-G1-C9 Q-disc stack. Two hundred microliters (exact mass was recorded) of the spike (deuterated PAHs) in toluene was added on top of the sample material. After a minimal exposure time of 30 min, the Q-cup was closed with a Q-screen before extraction. The sample was then extracted twice with 30 mL of a solvent mixture of methanol/tert-butyl methyl ether (tBME) (1:1, v/v) for 3 min at 120 °C.

The combined extracts were concentrated at 40 °C to 50 °C under an N_2_ stream to 2 mL. For solvent exchange, 10 mL of a mixture of cyclohexane/ethyl acetate (1:1, v/v) was added, followed by concentration at 40 °C to 50 °C under an N_2_ stream to 2 mL. The clean-up of the extract was adapted from the procedure described in [25] and was carried out in two steps. In the first step, a Supelclean EZ-POP NP (Merck KGaA, Darmstadt, Germany, Supelco® 54﻿341-U, bed A: 1.25 g Supelclean LC-Florisil; bed B: 1.25 g Z-Sep/C18, 12 mL) SPE cartridge was conditioned with 10 mL of cyclohexane and loaded with the concentrated extract (approx. 2 mL). After the extract had completely flowed into the stationary phase, the sample was eluted with 15 mL of cyclohexane. The eluate was then reduced to 1 mL at 40 °C to 50 °C under an N_2_ stream. In the second step, an AFFINIMIP SPE PAHs (Affinisep, Le Houlme, France, FS119-03-NG, 50 mg, 3 mL) SPE cartridge was conditioned with 3 mL of cyclohexane and loaded with the concentrated eluate from the first clean-up step (approx. 1 mL). The sample was washed with 1 mL of cyclohexane and eluted 3 times with 1 mL of ethyl acetate. The eluate was then carefully reduced to approx. 200 µL under an N_2_ stream at 40 °C to 50 °C and transferred to a GC vial. The collection tube was rinsed 2 times with 100 µL of toluene. The rinsing solution was combined with the concentrated eluate in the GC vial. After the addition of 100 µL (exact mass was recorded) of injection standard (FBkF) in toluene, the volume of the measurement solution was reduced under an N_2_ stream to approx. 0.5 mL.

#### Calibration blends

Six calibration blends in the range of 0.5 µg/kg to 8 µg/kg were prepared by mixing reference, spike, and injection standard in toluene. This six-point calibration was used for the quantification of the measured area ratios in the sample blends. The calibration functions for all investigated PAHs were assumed to be linear and were obtained by regression analysis. The evaluation of linearity was based on a “lack-of-fit” test in conjunction with a visual inspection of the residual plot [26].

#### GC–MS/MS measurement

The parameters for the GC–MS/MS measurements are summarized in Tables 1 and 2.

**Table 1:** GC–MS/MS instrumental parameters used for PAH analysis of WP-CBR001

Instrumental parameters	
GC system	Thermo Scientific Trace 1310
Pre-column	Phenomenex Zebron Guard Column, 5 m × 0.18 mm, deactivated
Column	Phenomenex ZB-PAH, 20 m × 0.18 mm, 0.14 µm
Injection	PTV (split, split flow = 5.0 mL/min, split ratio = 5, purge flow = 5.0 mL/min)
PTV program	60 °C (1 min) → (10 °C/s) 300 °C (10 min) → (1 °C/s) 325 °C (5 min)
Injection volume	1.5 µL
Oven program	60 °C (1 min) → (12 °C/min) 210 °C → (8 °C/min) 280 °C (9.75 min) → (8 °C/min) 320 °C (2 min)
Carrier gas (flow)	Helium (1.0 mL/min)
MS system	Thermo Scientific TSQ 8000 Evo
Transfer line temperature	300 °C
Ion source temperature	300 °C
Ionization	70 eV (EI)
Polarity	Positive
Acquisition mode	SRM

**Table 2:** GC–MS/MS parameters used for PAH measurements of WP-CBR001

			Quantification			Confirmation		
Compound	Sum formula	*RT*, ca. (min)	Precursor (*m/z*)	Fragment (*m/z*)	Collision energy (eV)	Precursor (*m/z*)	Fragment (*m/z*)	Collision energy (eV)
D_12_-BaA	C_18_D_12_	20.02	240.2	236.1	30	240.2	212.1	30
BaA	C_18_H_12_	20.10	228.1	226.1	30	228.1	202.1	20
D_12_-Chr	C_18_D_12_	20.25	240.2	236.1	30	240.2	212.2	30
Chr	C_18_H_12_	20.33	228.1	226.1	30	228.1	202.0	20
FBkF	C_20_H_11_F	22.97	270.1	268.0	30	270.1	250.1	30
D_12_-BbF	C_20_D_12_	23.17	264.2	260.1	30	264.2	236.2	30
BbF	C_20_H_12_	23.26	252.1	250.1	30	252.1	226.1	30
D_12_-BaP	C_20_D_12_	24.62	264.2	260.2	30	264.2	236.1	30
BaP	C_20_H_12_	24.72	252.1	250.1	30	252.1	225.9	30

### Homogeneity study

#### Between-unit homogeneity

The minimum number of bottles (units) for the between-unit homogeneity study was calculated in accordance with the recommendations given in Section 7.4.1 of ISO Guide 35:2017 [24] to be *N*_min_ = 10. Therefore, 10 bottles covering the whole bottling range of WP-CBR001 were randomly selected and stored at − 80 °C before analysis. Three independent test portions of each bottle were then analyzed. A total of 30 analyses were performed under repeatability conditions using the method described in “Analytical method” and according to a random sequence in order to prevent any possible trends in the filling order due to the analytical sequence. According to ISO Guide 35:2017 [24], the assessment of the between-unit homogeneity was carried out by a one-way analysis of variance (ANOVA).

#### Minimum sample amount

The minimum sample amount is closely related to the within-unit homogeneity. For all homogeneity, stability, and characterization measurements presented in this work, a sample amount of 3 g (see “Analytical method”) was used. Since some laboratories prefer sample amounts < 3 g, we investigated the minimum sample amount by repeated measurements (*n* = 6) of the material from the same bottle with sample amounts of 3 g and 1 g. To determine whether a similar accuracy can be obtained with a sample amount of 1 g as with 3 g, the two groups of results were compared to each other using an *F*-test for the comparison of the two variances and a *t*-test for the comparison of the two means. Furthermore, the results were compared to the certified values and their associated uncertainties.

### Stability study

For the stability study, an isochronous approach designed according to Section 8.2 of ISO Guide 35:2017 [24] was employed. The investigated bottles (one for each stability point) were stored for 1.5, 3, 6, and 12 months at different temperatures: − 20 °C, 4 °C, room temperature (approx. 20 °C), and 45 °C (up to 3 months only). After the storage time was reached for a certain stability point, the corresponding bottle was stored at the reference temperature (− 80 °C) before it was analyzed three times using the method described in “Analytical method.” For *t* = 0, data from the homogeneity study were used. The long-term stability, including 2-year and 4-year stability points, will be further investigated in the future.

### Characterization and value assignment

The assignment of the certified PAH mass fractions of WP-CBR001 was based on an in-house study at METAS analyzing the material using the GC-IDMS method described in “Analytical method”. For the certification, the data set of the homogeneity study was used. Uncertainties were assessed according to JCGM 100:2008 (GUM) [27] and with the software METAS UncLib implemented in Python [28].

### Verification of certified values

In order to verify the certified values, they were compared to the gravimetric mass fractions obtained from the production of the material and to the results of an ILC study. Both the gravimetric mass fractions and the results of the ILC were used for verification purposes only and were therefore not included in the value assignment.

#### Gravimetric mass fractions

The gravimetric mass fractions *w*_grav_(PAH) were determined from the masses of the materials used for the individual production steps. All masses were air buoyancy–corrected and uncertainties were assessed according to JCGM 100:2008 (GUM) [27] and using METAS UncLib implemented in Python [28].

#### Interlaboratory comparison

METAS and Merck organized an ILC study with selected laboratories as a proficiency test (PT). The participating laboratories were free to choose their analytical methods and the amount of sample for analysis. The ILC was managed by Merck as an independent party following ISO 17043 [29]. Neither METAS nor Merck contributed any analytical results to the ILC.

## Results and discussion

### Homogeneity assessment

#### Between-unit homogeneity

**Fig. 2:**
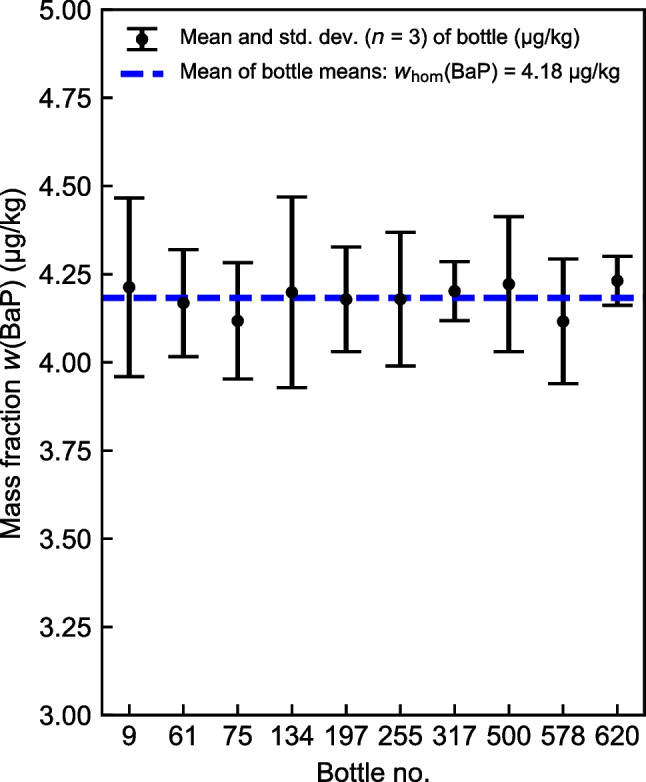
Homogeneity study of BaP: mean values of 10 selected bottles with their corresponding standard deviations (*n* = 3)

The results of the between-unit homogeneity assessment of all four PAHs are shown in Table 3. As an example, Fig. 2 illustrates the measurement results for BaP. The results are plotted in the order in which the bottles were filled. No trend was observed in the filling sequence. Illustrations for the other three PAHs and the raw data are given in Fig. S2 and Tables S17 to S20, respectively, in the Supplementary Information. The mean mass fraction of the homogeneity study is generally defined as the mean of bottle means. Because for each bottle (*i* = 1 to 10) an equal number of sample preparations/measurements (*j* = 1 to 3) were performed, *w*_hom_(PAH) was calculated as the mean value of all individual results according to Eq. 1.

**Table 3:** Analysis of variance (ANOVA) and estimates for uncertainty contribution due to potential inhomogeneity for BaA, BaP, BbF, and Chr

PAH	*w*_hom_(PAH) (µg/kg)	*F*_obs_	*F*_crit_	*s*_bb_ (µg/kg)	u*_bb_ (µg/kg)	*u*_hom_ (µg/kg)	*u*_hom, r_ (%)
BaA	3.1724	1.10	2.39	0.0154	0.0277	0.0277	0.872
BaP	4.1826	0.15	2.39	n/a	0.0586	0.0586	1.400
BbF	4.7291	0.22	2.39	n/a	0.0497	0.0497	1.051
Chr	2.8471	1.05	2.39	0.0120	0.0300	0.0300	1.053

1 $${w}_{\mathrm{hom}}\left(\mathrm{PAH}\right)=\frac{1}{n}\bullet \frac{1}{k}\bullet \sum_{i=1}^{n}\sum_{j=1}^{k}{w}_{\mathrm{hom},ij}\left(\mathrm{PAH}\right)$$

The mean squared deviations between and within bottles (*MS*_between_ and *MS*_within_) were obtained from one-way ANOVA. The observed *F*-value, *F*_obs_, was calculated as the ratio *MS*_between_/*MS*_within_ and the critical *F*-value, *F*_crit_, was obtained from the *F*-value table applying a significance level *α* = 0.05. For all four PAHs, *F*_obs_ were lower than *F*_crit_, indicating that the variances of the measured values within and between the bottles do not differ significantly at a 95% confidence level. No evidence of statistically significant inhomogeneity was therefore observed. The uncertainty contributions for potential inhomogeneities *u*_hom_[*w*_hom_(PAH)] were estimated using Eqs. 2 and 3 for *s*_bb_[*w*_hom_(PAH)] and u*_bb_[*w*_hom_(PAH)], respectively, following the guidelines of ISO Guide 35:2017 [24] and Linsinger et al. [30]. For the estimation of *u*_hom_[*w*_hom_(PAH)], the higher value of *s*_bb_[*w*_hom_(PAH)] and u*_bb_[*w*_hom_(PAH)] was taken.

2 $${s}_{\mathrm{bb}}\left[{w}_{\mathrm{hom}}\left(\mathrm{PAH}\right)\right]=\sqrt{\frac{{MS}_{\mathrm{between}}-{MS}_{\mathrm{within}}}{n}}$$

3 $${u}_{\mathrm{bb}}^{*}\left[{w}_{\mathrm{hom}}\left(\mathrm{PAH}\right)\right]=\sqrt{\frac{{MS}_{\mathrm{within}}}{n}}\bullet \sqrt[4]{\frac{2}{{v}_{{\mathrm{MS}}_{\mathrm{within}}}}}$$

*n:* number of replicate determinations for each unit (*n* = 3)

*v*_*MSwithin*_: degrees of freedom of *MS*_within_ (${v}_{{MS}_{within}}$ = 20)

#### Minimum sample amount

The results, which are reported in detail in Table S1 and Fig. S3 in the Supplementary Information, show that the mean and standard deviations of the replicate measurements were comparable for sample amounts of 1 g and 3 g. For all investigated PAHs, the *F*- and *t*-tests indicated that there is no evidence of a significant difference between the variances and the means of the two groups of results at the 95% level of confidence. The expanded uncertainties of the certified values (see “Uncertainty budget”) are larger by a factor of two or more than the expanded standard deviations (2* s*) resulting from the sample amount study. A sample amount of 1 g yielded acceptable accuracy and is therefore recommended as the minimum sample amount.

### Stability assessment

According to ISO Guide 35:2017 [24] and Linsinger et al. [30], the stability was assessed by applying a linear regression model (see Eq. 4), where the slope *b*_1_ and intercept *b*_0_ were fitted to the stability data.

4 $$w_{\mathrm{lts}\;\mathrm{or}\;\mathrm{sts}}\left(\mathrm{PAH}\right)=b_1\bullet t_{\mathrm{storage}}+b_0$$

The raw data are listed in Tables S21 to S24 in the Supplementary Information. A two-tailed *t*-test showed that the slopes for all four PAHs at all investigated temperatures did not differ significantly from 0 at a 95% confidence level (see Tables 4 and 5 for the assessments at temperatures of − 20 °C and 45 °C, respectively). The *t*-value, *t*_b1_, was calculated as |*b*_1_|/*s*(*b*_1_), with the standard uncertainty of the slope, *s*(*b*_1_), obtained from regression analysis. The critical *t*-value, *t*_crit_, was obtained from the *t*-value table (two-tailed, significance level *α* = 0.05, degrees of freedom = *n* – 2).

**Table 4:** Results of the two-tailed *t*-test of the long-term stability at − 20 °C for WP-CBR001

PAH	*b*_1_ (µg × kg^−1^ × months^−1^)	*b*_0_ (µg × kg^−1^)	*s*(*b*_1_) (µg × kg^−1^ × months^−1^)	*t*_b1_	*t*_crit_
BaA	− 0.004019	3.170441	0.006141	0.654	2.160
BaP	− 0.005942	4.248284	0.008734	0.680	2.160
BbF	0.007616	4.847790	0.009581	0.795	2.160
Chr	− 0.006308	2.882507	0.006140	1.027	2.160

**Table 5:** Results of the two-tailed *t*-test of the short-term stability at 45 °C for WP-CBR001

PAH	*b*_1_ (µg × kg^−1^ × months^−1^)	*b*_0_ (µg × kg^−1^)	*s*(b_1_) (µg × kg^−1^ × months^−1^)	*t*_b1_	*t*_crit_
BaA	− 0.017971	3.198704	0.023153	0.776	2.364
BaP	− 0.003442	4.179824	0.028087	0.123	2.364
BbF	0.018400	4.772878	0.025946	0.709	2.364
Chr	0.024231	2.829729	0.015095	1.605	2.364

Since no evidence of statistically significant instability was found at the various temperatures during the investigated storage times, *b*_1_ was set to 0 for further calculations. The intercept *b*_0_(*b*_1_ = 0) and the standard uncertainty of the slope *s*(*b*_1_ = 0) were then calculated according to Eqs. 5 and 6, respectively.

5 $$b_0\left(b_1=0\right)=w_{\mathrm{lts}\;\mathrm{or}\;\mathrm{sts}}\left(\mathrm{PAH}\right)=\frac1n\bullet\frac1k\bullet\sum_{i=1}^n\sum_{j=1}^kw_{\mathrm{lts}\;\mathrm{or}\;\mathrm{sts},\;ij}\left(\mathrm{PAH}\right)$$

6 $$s\left(b_1=0\right)=\frac{s\left[w_{\mathrm{lts}\;\mathrm{or}\;\mathrm{sts}}\left(\mathrm{PAH}\right)\right]}{\sqrt{\sum\left(t_{\mathrm{storage},i}-{\overline t}_{\mathrm{storage}}\right)^2}}$$

For the estimation of the uncertainties for potential long-term, *u*_lts_[*w*_lts_(PAH)], and short-term, *u*_sts_[*w*_sts_(PAH)], instabilities, storage temperatures of − 20 °C and 45 °C, respectively, were applied. The uncertainties were calculated according to the extrapolation model given in Eq. 7 [24, 30], using storage times of 24 months and 0.5 months (2 weeks) for long- and short-term stabilities, respectively, and are listed in Tables 6 and 7.

**Table 6:** Analysis of the long-term stability at − 20 °C for WP-CBR001

PAH	*s* (*b*_1_ = 0) (µg·kg^−1^·months^−1^)	*b*_0_ (*b*_1_ = 0) = *w*_lts_(PAH) (µg/kg)	*u*_lts_[*w*_lts_(PAH)] (µg/kg)	*u*_lts, r_[*w*_lts_(PAH)] (%)
BaA	0.006014	3.152356	0.144	4.579
BaP	0.008565	4.221544	0.206	4.869
BbF	0.009455	4.882063	0.227	4.648
Chr	0.006152	2.854121	0.148	5.173

**Table 7:** Analysis of the short-term stability at 45 °C for WP-CBR001

PAH	*s* (*b*_1_ = 0) (µg·kg^−1^·months^−1^)	*b*_0_ (*b*_1_ = 0) = *w*_sts_(PAH) (µg/kg)	*u*_sts_[*w*_sts_(PAH)] (µg/kg)	*u*_sts, r_[*w*_sts_(PAH)] (%)
BaA	0.022571	3.171747	0.011	0.356
BaP	0.026302	4.174661	0.013	0.315
BbF	0.025127	4.800478	0.013	0.262
Chr	0.016516	2.866075	0.008	0.288

7 $$u_{\mathrm{lts}\;\mathrm{or}\;\mathrm{sts}}\left[w_{\mathrm{lts}\;\mathrm{or}\;\mathrm{sts}}\left(\mathrm{PAH}\right)\right]=s\left(b_1=0\right)\cdot t_{\mathrm{storage}}$$

**Fig. 3:**
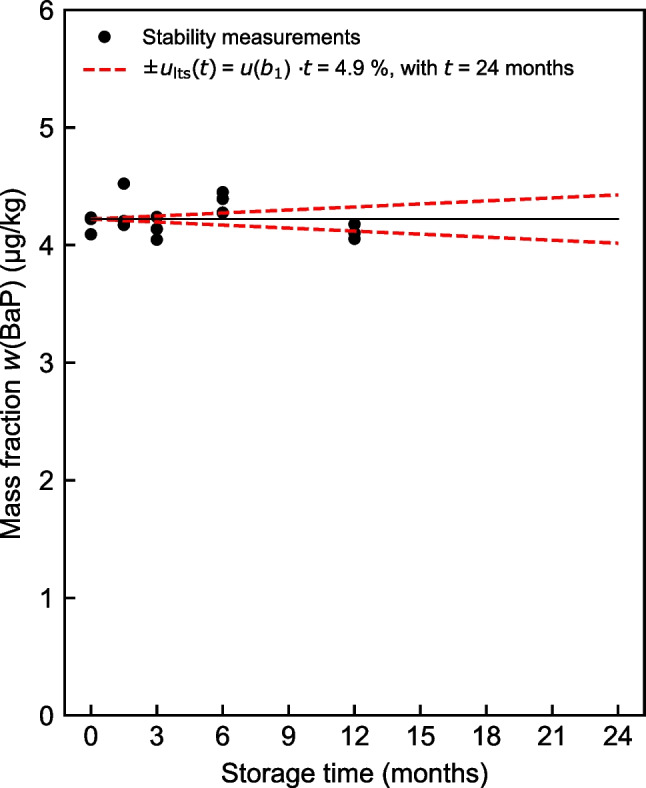
Long-term stability of BaP at − 20 °C with estimated relative standard uncertainty *u*_lts_

Figure 3 shows the extrapolation results for the long-term stability study at − 20 °C for BaP. For the other three PAHs BaA, BbF, and Chr as well as for the short-term stability, the results are illustrated in Figs. S4 and S7 in the Supplementary Information.

For the assessment of long-term stability, storage temperatures at 4 °C and room temperature (approx. 20 °C) resulted in uncertainty estimates similar to those at − 20 °C, showing that the long-term stability would also be given at temperatures up to room temperature (see Figs. S5 and S6 in the Supplementary Information).

For short-term stability, the estimated uncertainties are all < 0.5%, clearly below the ones for long-term stability. An unintentional exposure to higher temperatures for a short amount of time, e.g., during transportation, would therefore not have any significant effect on the material.

### Characterization

For the characterization of WP-CBR001, the measurement data of the homogeneity assessment were used. Since the sample preparation steps described in “Analytical method” are very time-consuming, the analyses had to be performed over three different days (10 samples per day). Therefore, day-to-day variations are taken into account in the evaluation of the characterization. However, no trend in the analysis sequence could be identified across all 30 sample work-ups and measurements. From our experience, the main contribution to the spread of the measurement results is mainly from the extraction step of the material. The individual measurement values are given in Tables S17 to S20 in the Supplementary Information. For each PAH, the mass fraction of the characterization was calculated as *w*_char_(PAH) = *w*_hom_(PAH) according to Eq. 1.

For the estimation of the standard uncertainty of the characterization process, *u*_char_[*w*_char_(PAH)], the contributions of the measurement steps, *u*_meas_[*w*_char_(PAH)], and the repeatability, *u*_rep_[*w*_char_(PAH)], were assessed. Both contributions were then combined to *u*_char_[*w*_char_(PAH)] according to Eq. 8.

8 $${u}_{\mathrm{char}}\left[{w}_{\mathrm{char}}\left(\mathrm{PAH}\right)\right]=\sqrt{{u}_{\mathrm{meas}}^{2}\left[{w}_{\mathrm{char}}\left(\mathrm{PAH}\right)\right]+{u}_{\mathrm{rep}}^{2}\left[{w}_{\mathrm{char}}\left(\mathrm{PAH}\right)\right]}$$

A detailed description of the measurement uncertainty assessment is given in chapters 5 to 8 in the Supplementary Information. The mass fractions *w*_char_(PAH) and their associated standard uncertainties *u*_char_[*w*_char_(PAH)] are given in Table 8.

**Table 8:** Mass fractions *w*_char_(PAH) and estimated standard uncertainties *u*_char_[*w*_char_(PAH)] of the characterization

PAH	*w*_char_(PAH) (µg/kg)	*u*_char_[*w*_char_(PAH)] (µg/kg)	*u*_char,r_[*w*_char_(PAH)] (%)
BaA	3.1724	0.0536	1.69
BaP	4.1826	0.1080	2.58
BbF	4.7291	0.0933	1.97
Chr	2.8471	0.0572	2.01

### Uncertainty budget

The combined uncertainty was calculated using Eq. 9 by considering the relative standard uncertainty contributions from the characterization of the material, *u*_char,r_[*w*_char_(PAH)], the homogeneity assessment, *u*_hom,r_[*w*_hom_(PAH)], and the short- and long-term stability assessments, *u*_sts,r_[*w*_sts_(PAH)] and *u*_lts,r_[*w*_lts_(PAH)].

9 $${u}_{\mathrm{c}}\left[{w}_{\mathrm{char}}\left(\mathrm{PAH}\right)\right]={w}_{\mathrm{char}}\left(\mathrm{PAH}\right)\bullet \sqrt{\begin{array}{c}{u}_{\mathrm{char},\mathrm{r}}^{2}\left[{w}_{\mathrm{char}}\left(\mathrm{PAH}\right)\right]+{u}_{\mathrm{hom},\mathrm{r}}^{2}\left[{w}_{\mathrm{hom}}\left(\mathrm{PAH}\right)\right]\\ +{u}_{\mathrm{Its},\mathrm{r}}^{2}\left[{w}_{\mathrm{lts}}\left(\mathrm{PAH}\right)\right]+{u}_{\mathrm{sts},\mathrm{r}}^{2}\left[{w}_{\mathrm{sts}}\left(\mathrm{PAH}\right)\right]\end{array}}$$

The expanded uncertainties were calculated using Eq. 10 based on the combined uncertainties applying a coverage factor *k* = 2.

10 $$U\left[{w}_{\mathrm{char}}\left(\mathrm{PAH}\right)\right]={u}_{\mathrm{c}}\left[{w}_{\mathrm{char}}\left(\mathrm{PAH}\right)\right] \bullet k$$

As an example, the uncertainty budget of BaP is shown in Table 9. The uncertainty budgets of the other PAHs BaA, BbF, and Chr are given in Tables S10 to S12 in the Supplementary Information.

**Table 9:** Mass fractions and estimated combined and expanded uncertainties of BaP in WP-CBR001. *Contribution to combined standard uncertainty. **Percentage contribution of *u*_*i*_^2^[*w*_char_(BaP)] to *u*_c_^2^[*w*_char_(BaP)]

BaP	Contribution *i*			
	Characterization (char)	Homogeneity (hom)	Long-term stability (lts)	Short-term stability (sts)
*w*_*i*_(BaP) (µg/kg)	4.1826	4.1826	4.2215	4.1766
*u*_*i*_[*w*_*i*_(BaP) (µg/kg)	0.1080	0.0586	0.2056	0.0132
*u*_*i*,r_[*w*_*i*_(BaP)] (-)	0.0258	0.0140	0.0487	0.0032
*u*_*i*_[*w*_char_(BaP)]* (µg/kg)	0.1080	0.0586	0.2037	0.0132
*%u*_c_[*w*_char_(BaP)]** (%)	20.6	6.0	73.1	0.3
*u*_c_[*w*_char_(BaP)] (µg/kg)	0.2382			
*U*[*w*_char_(BaP)] (µg/kg)	0.48			

### Certified values and metrological traceability

The certified mass fractions, *w*_char_(PAH), of BaA, BaP, BbF, and Chr, given in Table 10, are based on the in-house certification results obtained by the characterization of the material. The measured value, *w*_char_(PAH), and the associated expanded uncertainty, *U*[*w*_char_(PAH)], represent the interval, *w*_char_ ± *U*[*w*_char_(PAH)], which contains the value of the measured quantity with a probability of approximately 95%.

**Table 10:** Certified mass fractions of BaA, BaP, BbF, and Chr in WP-CBR001

PAH	Mass fraction (µg/kg)	
	Certified value	*U*
Benz[*a*]anthracene BaA	3.17	0.32
Benzo[*a*]pyrene BaP	4.18	0.48
Benzo[*b*]fluoranthene BbF	4.73	0.49
Chrysene Chr	2.85	0.33

Intermediate results were not rounded. Rounding was done for the expanded uncertainties, not for the combined uncertainties. Uncertainties were always rounded up. The measurement results were rounded up or down according to the usual rounding rules.

The reported measurement values are traceable to national standards and thus to internationally supported realizations of the SI units. All certified values refer to the mass fractions of BaA, BaP, BbF, and Chr. In order to ensure metrological traceability of the mass fractions as defined above, the gravimetrically prepared certified calibration standard SRM 1647f (NIST, Gaithersburg, USA) was employed for the in-house certification study and taken into account for the assessment of *u*_char_[*w*_char_(PAH)], the uncertainty of the mass fractions obtained in the characterization step. Traceability was further established by using GC linear regression IDMS measurements.

### Verification of certified values

#### Gravimetric mass fractions

**Fig. 4:**
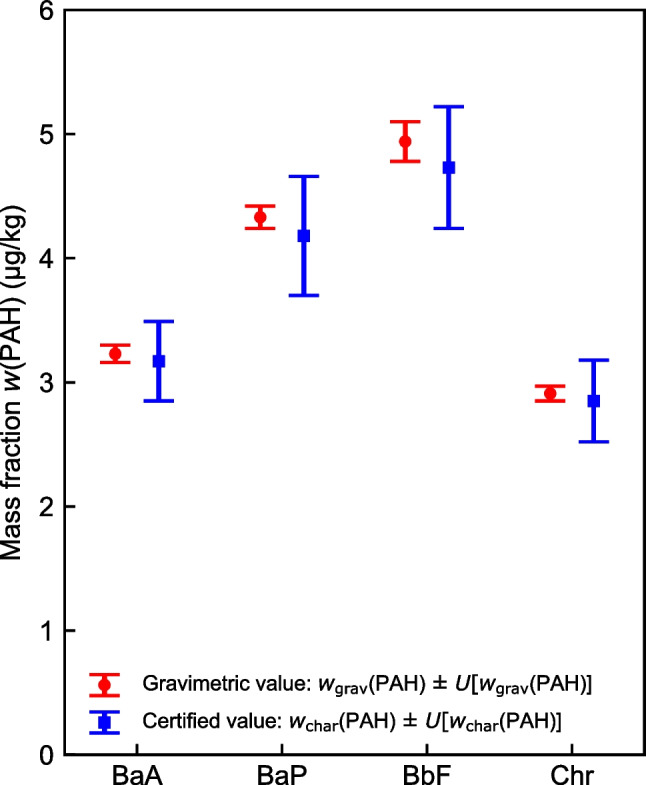
Comparison of the certified values and their associated expanded uncertainties with the values obtained from gravimetric production of WP-CBR001

In Fig. 4, the certified values, which are obtained from characterization, *w*_char_(PAH), are compared to the gravimetric mass fractions, *w*_grav_(PAH), which are listed in Table 11. Calculation details and input values for the determination of the gravimetric mass fractions and their associated expanded uncertainties are given in Tables S13 to S16 in the Supplementary Information. For all four PAHs, the expanded uncertainties of the certified values, *U*[*w*_char_(PAH)], show a good overlap with the expanded uncertainties estimated for the gravimetric mass fractions, *U*[*w*_grav_(PAH)], supporting our certification approach.

**Table 11:** Gravimetric mass fractions and expanded uncertainties (*k* = 2) of BaA, BaP, BbF, and Chr in WP-CBR001

PAH	*w*_grav_(PAH) (µg/kg)	*U*[*w*_grav_(PAH)], *k* = 2 (µg/kg)	*U*_r_[*w*_grav_(PAH)], *k* = 2 (%)
BaA	3.23	0.07	2.2
BaP	4.33	0.09	2.1
BbF	4.94	0.16	3.3
Chr	2.91	0.06	2.1

#### Interlaboratory comparison study

The ILC study, organized as a proficiency test (PT), was intended as an additional verification of the certified values. The results were not included in the calculations of the certified values and their uncertainties. Only laboratories known to have long-standing experience in the analysis of PAHs in food were asked to participate in the ILC during a defined period of time. Ultimately, twelve official control laboratories from Switzerland and Germany participated in the ILC round PE5008-30G (METAS21-3). Although the number of participants was rather small, the careful selection of the laboratories and their commitment to detailed discussions after the ILC formed a good basis for deriving important information from the study.

**Fig. 5:**
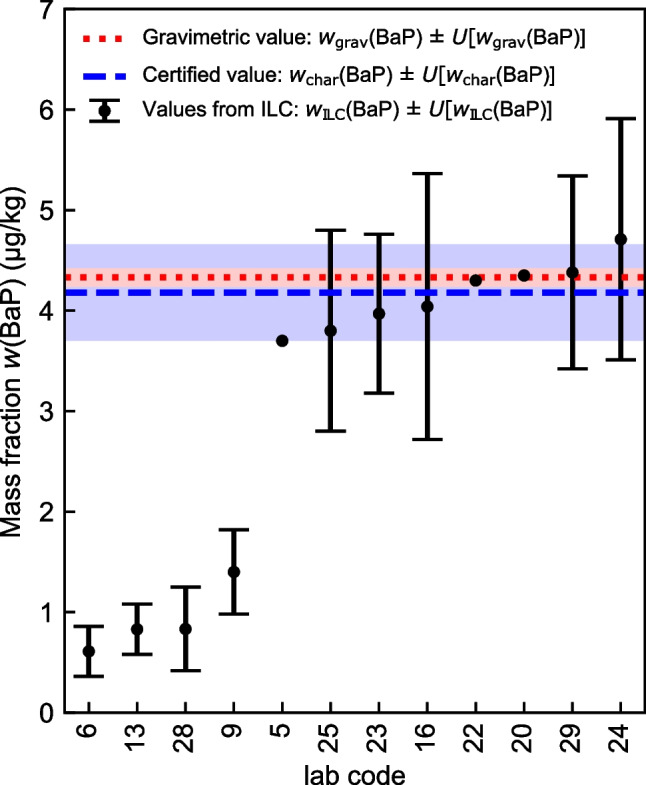
Comparison of the certified mass fractions of BaP with the results of the ILC study. For data points without uncertainty bars, no uncertainty values were submitted by the corresponding laboratories

In Fig. 5, the mass fractions and expanded uncertainties for BaP obtained by the certification study of WP-CBR001 are compared to the results of the ILC. The results of BaA, BbF, and Chr, which are given in Fig. S11 in the Supplementary Information, are very similar to the ones of BaP. Note that the results of the ILC are given based on a dry mass basis whereas the certified values of WP-CBR001 are given based on the material as is. Using the reported results of dry weight contents of the material from the participating laboratories, a value of 0.9587 g/g could be calculated from algorithm A according to ISO 13528 [31], chapter C3.1. The mass fractions of the participating laboratories shown in Fig. 5 are therefore about 4% higher than if they had been reported on the material as is.

Although the data basis is not very large, visual inspection of the graphical evaluation (see Fig. 5 for BaP) reveals a tendency towards a bimodal distribution of the data for all four PAHs. While for each PAH the main mode (including the eight laboratories 5, 25, 23, 16, 22, 20, 29, and 24) was located near the gravimetric mass fractions and the certified values, the sub-mode (including the four laboratories 6, 13, 28, and 9) was located at mass fractions that were up to ten times lower. Evaluation of additional method information from the participants of the ILC revealed that laboratories belonging to the sub-mode performed direct extractions of the sample material using mixtures of n-hexane/acetone and cyclohexane/ethyl acetate. In contrast, laboratories belonging to the main mode used methanol/tBME mixtures for direct extraction, applied different extraction techniques such as QuEChERS, or treated the sample by saponification with methanolic KOH before subjecting it to liquid–liquid extraction with cyclohexane. A dependence on the measurement technique was not evident. Results from both modes were obtained by GC–MS and LC-FLD, in accordance with the findings of Sykes and co-workers [32].

To investigate the extraction efficiency of different solvents and solvent mixtures in more detail, a systematic study was conducted. First, the study included solvents and solvent mixtures (n-hexane, cyclohexane, cyclohexane/acetone, cyclohexane/ethyl acetate) that are frequently used in the extraction step of PAHs analysis.

Second, methanol and mixtures with tBME (1:1 and 1:4, v/v) that are used for the extraction of other process contaminants (2- and 3-monochloropropanediol, glycidyl esters) in similar types of matrices [33] were added to the study. While the solvent varied, the extraction parameters of the ASE (EDGE) extraction system (see “Analytical method”) were kept constant.

The study was extended by comparing different extraction procedures. Besides ASE (EDGE) extraction, a modified QuEChERS [34] and a saponification (methanolic KOH) [35] procedure were tested. The main steps of the QuEChERS procedure consisted of soaking the sample in water before adding a mixture of hexane/acetone (1:1, v/v) or acetonitrile and the unbuffered QuEChERS salt (4 g MgSO4 and 1 g NaCl) for extraction, whereas the main step in the saponification procedure consisted of extraction of the saponified sample with n-hexane.

In all experiments of the study, only the extraction step was varied, while all other steps of the method (see “Analytical method”) were maintained, including the addition of the deuterated PAHs before extraction, clean-up of the extracts, and the measurement by GC–MS/MS.

**Fig. 6:**
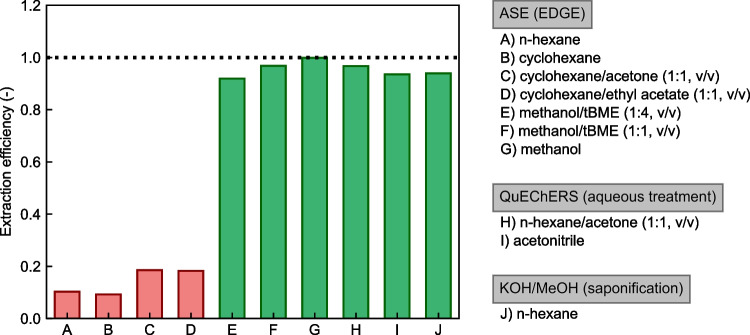
Solvent extraction efficiencies of BaP for WP-CBR001

The results of the solvent extraction efficiency assessment for BaP in WP-CBR001 are shown in Fig. 6. The results for BaA, BbF, and Chr are given in Fig. S12 in the Supplementary Information. The extraction efficiencies were calculated as the ratio between the mass fraction of the corresponding PAH obtained by the experiment applied and the gravimetric mass fraction (*w*_grav_) calculated from production data. Experiments A to I were performed with WP-CBR001 and experiment J with a laboratory test material with the same properties as WP-CBR001 except for slightly different gravimetric mass fractions of the PAHs. For all four PAHs, when using ASE with the nonpolar extraction solvents n-hexane and cyclohexane, as specified, for example, in EN 16619:2015 [36], the resulting extraction efficiencies were at around 0.1 (experiments A and B). Even when the polarity was increased by adding acetone or ethyl acetate to cyclohexane (experiments C and D), the measured extraction efficiencies remained below 0.2. On the contrary, when the ASE extractions of the samples were performed with methanol or mixtures of methanol with tBME (experiments E to G), the measured extraction efficiencies of all four PAHs drastically increased to values close to 1.

Extraction efficiencies close to 1 were also obtained for all four PAHs with the QuEChERS and the saponification procedures (experiments H to J). These results show that the crucial step of the sample preparation is the selection of the right extraction solvent rather than the extraction procedure itself. Although only a limited number of solvents and extraction procedures were tested, it is evident that a polar protic solvent like methanol or water (used to soak the whey protein powder) is the key to the efficient extraction of PAHs from the whey protein matrix. Our results demonstrate that nonpolar (n-hexane, cyclohexane) and polar aprotic solvents (acetone, ethyl acetate), which are often used for PAH extraction, are too weak to create complete access to the PAHs in this kind of matrix. Even the addition of deuterated internal standards prior to the extraction step cannot correct for the poor extraction yield because they do not experience the same environment as the native components incorporated into the matrix, when only apolar or polar aprotic solvents are used for the extraction.

Based on the additional information on the analytical methods used by the participating laboratories and the results of our extraction study presented above, the low values of the laboratories belonging to the sub-mode can be attributed to the poor solvent extraction efficiency. Taking only the results belonging to the main mode into consideration, the mass fractions obtained by the certification study are in good agreement with the results of the laboratories participating in the ILC.

## Conclusions

WP-CBR001 is a whey-protein CRM developed for the determination of the PAHs BaA, BaP, BbF, and Chr in a protein-rich matrix.

For the production of this CRM, a whey protein matrix was chosen, as the latter is a product frequently used in the food industry. A sample of industrially produced whey was spiked with the contaminants and was subsequently spray-dried in an industrial pilot plant, allowing the contaminants to integrate the whey protein matrix. The final CRM consequently represents a material that is very close to a potentially contaminated food matrix rich in proteins.

The stability and the homogeneity of the PAHs in the produced CRM were assessed through systematic studies according to the ISO Guide 35:2017 [24]. The minimum sample amount needed for the analysis of the PAH mass fractions was defined as 1 g of the CRM WP-CBR001.

The bimodal results of the ILC and our study about the extraction efficiency demonstrated the importance of the right solvent choice. The results showed that for this whey protein matrix, only polar and protic solvents, as methanol or water, were able to provide access for complete extraction of the PAHs. On the other hand, nonpolar and polar aprotic solvents, such as n-hexane or ethyl acetate, seemed unable to extract the complete load of PAHs from this kind of matrix.

The certified mass fractions and expanded uncertainties of the PAHs in the CRM were (3.17 ± 0.32) µg/kg BaA, (4.18 ± 0.48) µg/kg BaP, (4.73 ± 0.49) µg/kg BbF, and (2.85 ± 0.33) µg/kg Chr. These values were verified by an ILC study and by the gravimetric mass fractions obtained from production data.

The protection of consumers from the intake of process contaminants exceeding health standards is an important task of food safety laboratories. As the required extraction methods vary significantly between different food matrices, the choice of a matrix CRM close to the studied material is crucial. In this article, we demonstrated that WP-CBR001 is a matrix CRM suitable for the development, validation, and performance control of analytical methods for the determination of PAHs in high-protein food matrices.

## Supplementary Information

Below is the link to the electronic supplementary material.

Supplementary file1 (PDF 1.54 MB)
