# Supplementary material for: Development of a novel certified reference material for the determination of polycyclic aromatic hydrocarbons (PAHs) in whey protein powder
Source: Anal Bioanal Chem. 2023 Jul 28;415(23):5819–32. doi: 10.1007/s00216-023-04863-9 (PMC10474187; doi:10.1007/s00216-023-04863-9)
Supplement: Supplementary file 1 — Supplementary file1 (PDF 1.54 MB) [file 216_2023_4863_MOESM1_ESM.pdf]

Supplementary Information for

"Development of a novel certified reference material for the determination of polycyclic aromatic hydrocarbons (PAHs) in whey protein powder"

Simon Lobsiger[1], Lena Märki[1], Silvia Mallia[1], Gisela Umbricht[1], Hanspeter Sprecher[2], Kathrin Breitruck[2], Markus Obkircher[3]

[1] Federal Institute of Metrology METAS, Lindenweg 50, 3003 Bern-Wabern, Switzerland

[2] Sigma-Aldrich Production GmbH (a subsidiary of Merck KGaA, Darmstadt, Germany), Industriestrasse 25, 9471 Buchs, Switzerland

[3] Sigma-Aldrich Chemicals Pvt Ltd (a subsidiary of Merck KGaA, Darmstadt, Germany), Plot No-12, Bommasandra Jigani Link Rd, Bengaluru, Karnataka 560099, India

Corresponding author: Simon Lobsiger (simon.lobsiger@metas.ch)

1. Production

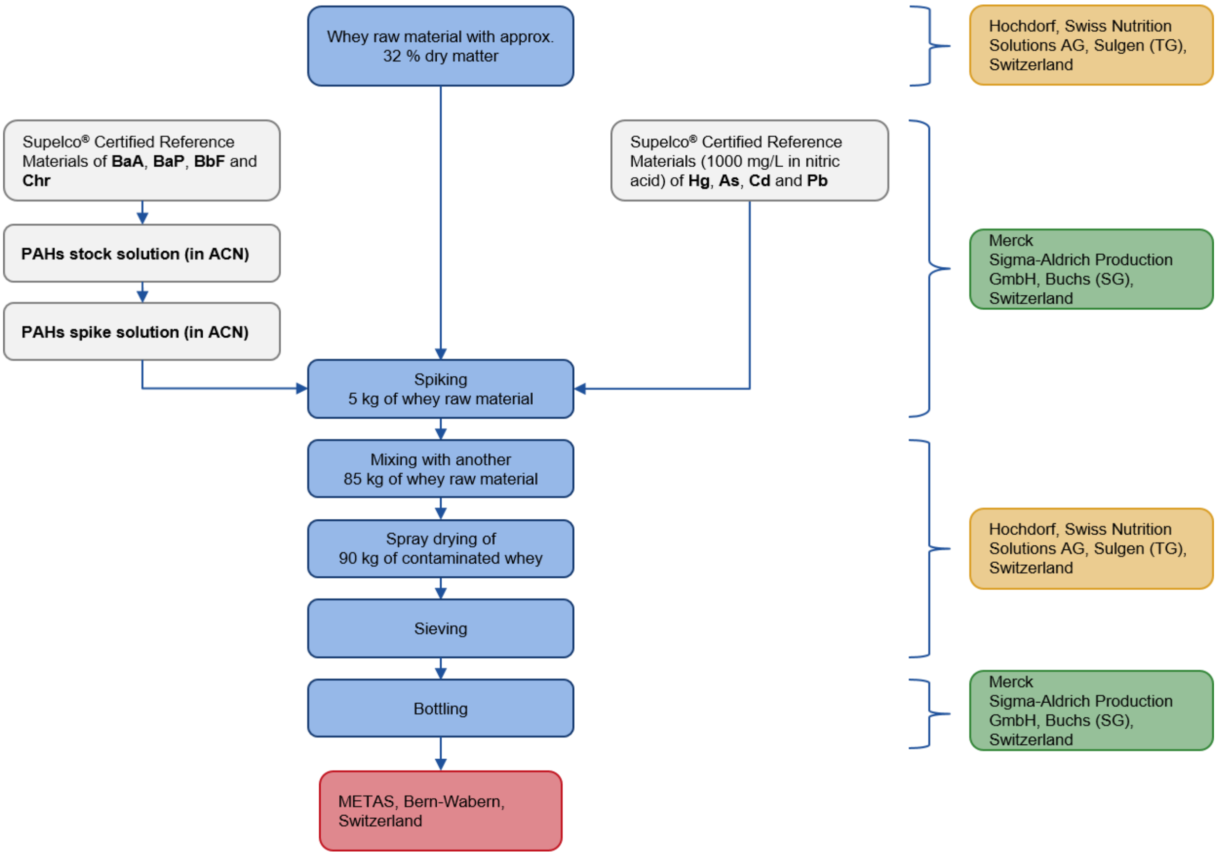

Fig. S1 Schematic representation of the production steps of the whey protein powder WP-CBR001

2. Homogeneity assessment

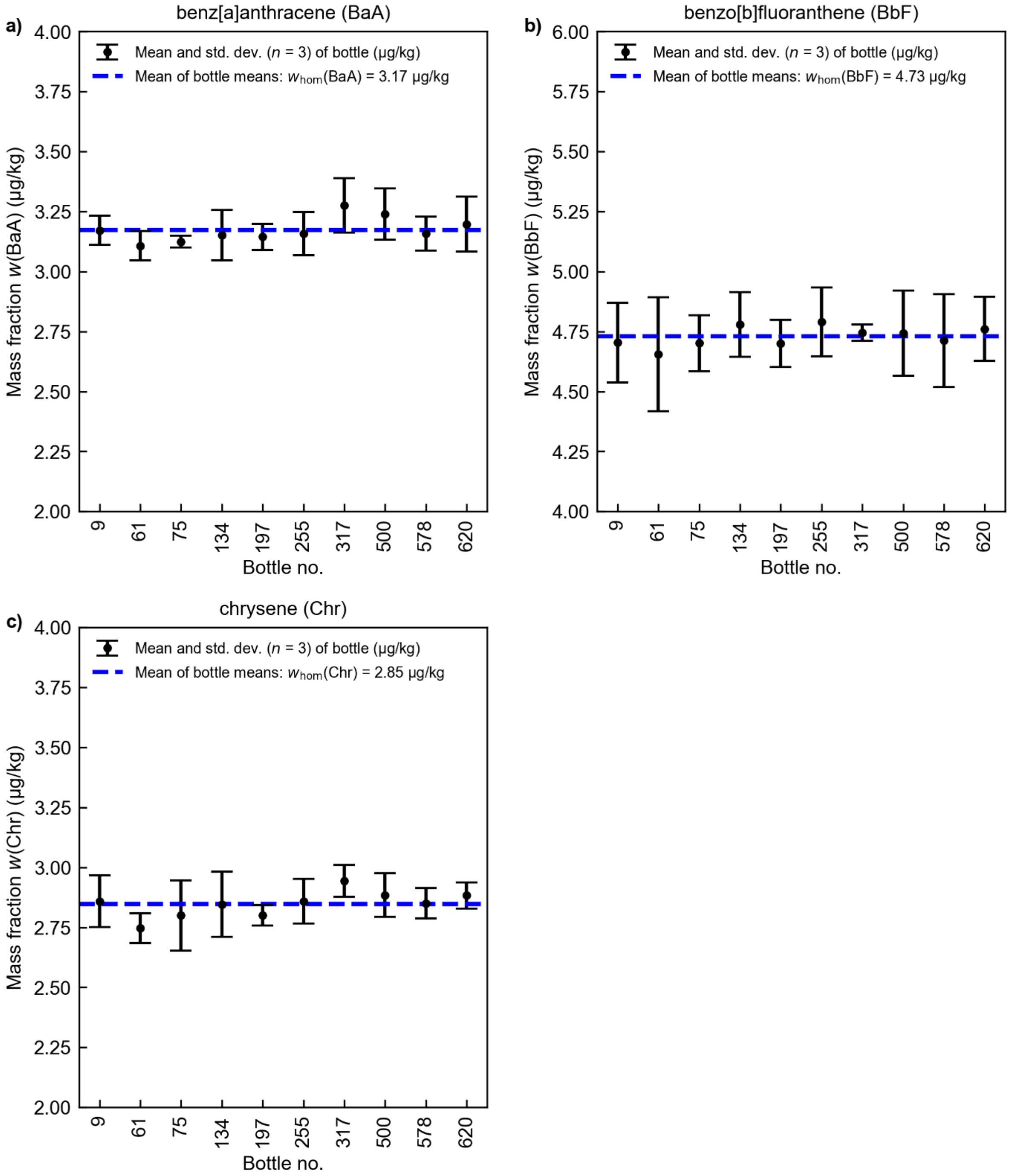

Fig. S2 Homogeneity study of BaA, BbF and Chr: mean values of 10 selected bottles with their corresponding standard deviations (n = 3)

3. Minimum sample size

Table S1 Comparison of the variances and the mean values of the four PAHs using sample amounts of 1 g and 3g. * The nominator and denominator for calculation F are chosen in a way that $F \geq 1$

Bottle no. 274	Mass fraction $w_{sa,1g}$(PAH)							
PAH	BaA		BaP		BbF		Chr	
Sample amount	1 g	3 g	1 g	3 g	1 g	3 g	1 g	3 g
1	3.2188	3.1869	4.3570	4.3291	4.7951	4.5908	3.0290	2.8758
2	3.2947	3.2523	4.4110	4.4216	4.8995	4.7800	2.9785	3.0007
3	3.1819	3.2189	4.2705	4.2502	4.7870	4.6659	3.0293	2.9599
4	3.2107	3.2998	4.3982	4.2964	4.8642	4.7125	3.0179	2.9714
5	3.0616	3.2645	4.1612	4.2947	4.5991	4.5382	2.9359	2.9483
6	3.2483	3.3592	4.4602	4.4200	4.9379	4.7691	2.9901	3.0514
n	6	6	6	6	6	6	6	6
Mean value $w_{sa,1g\ or\ 3g}$(PAH) (µg/kg)	3.2027	3.2636	4.3430	4.3353	4.8138	4.6761	2.9968	2.9679
Standard deviation $s_{sa,1g\ or\ 3g}$(PAH) (µg/kg)	0.0790	0.0607	0.1094	0.0708	0.1204	0.0971	0.0364	0.0583
Relative standard deviation $s_{sa,1g\ or3g,\ r}$(PAH) (%)	2.5	1.9	2.5	1.6	2.5	2.1	1.2	2.0
F-test (two-tailed) for the comparison of two variances								
H_0	σ^2(1 g) = σ^2(3 g)							
H_a	σ^2(1 g) ≠ σ^2(3 g)							
$F = (s_{sa,1g})^2/(s_{sa,3g})^2$*	0.592		0.418		0.651		2.562	
dof (n-1)	5	5	5	5	5	5	5	5
α	0.05							
$F_{crit,\ low}(1\text{-}\alpha/2,\ n_{sa,1g}\text{-}1,\ n_{sa,3g}\text{-}1)$	0.140							
$F_{crit,\ high}(\alpha/2,\ n_{sa,1g}\text{-}1,\ n_{sa,3g}\text{-}1)$	7.146							
Assessment	$F > F_{crit,\ low}$; $F < F_{crit,\ high}$ → no evidence for a significant difference between the two variances at the 95 % confidence level for all investigated PAHs							
t-test for the comparison of two means								
H_0	μ(1 g) = μ(3 g)							
H_a	μ(1 g) ≠ μ(3 g)							
s_{pooled}	0.0705		0.0922		0.1094		0.0486	
$\lvert t \rvert$	1.498		0.145		2.181		1.028	
dof (n-1)	5	5	5	5	5	5	5	5
α	0.05							
v	10		10		10		10	
$t_{crit}(1\text{-}\alpha/2,\ v)$	2.228							
Assessment	$\lvert t \rvert < t_{crit}$ → no evidence for a significant difference between the two means at the 95 % confidence level for all investigated PAHs							

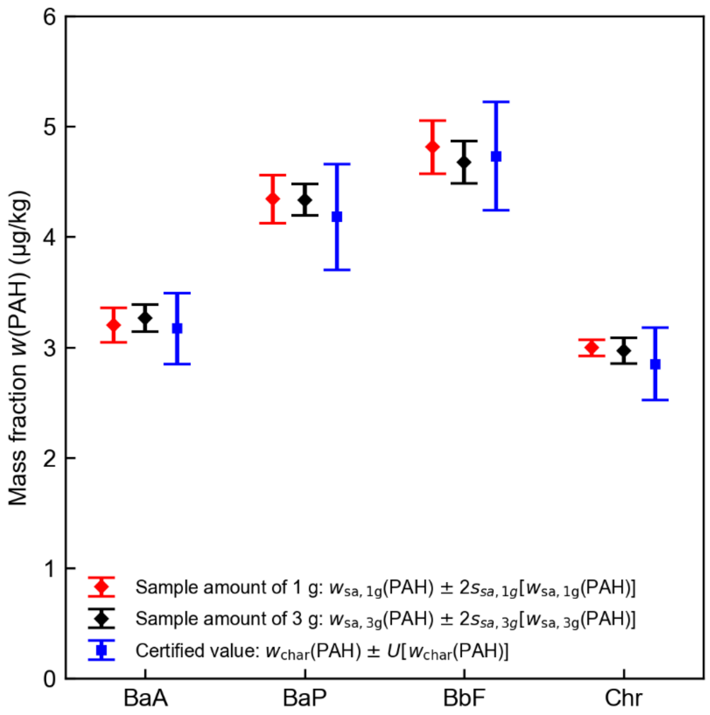

Fig. S3 Comparison of the mean values and expanded standard deviations (2*s*) obtained by sample amounts of 1 g and 3 g used for analysis compared to the certified values and expanded uncertainties

4. Stability assessment

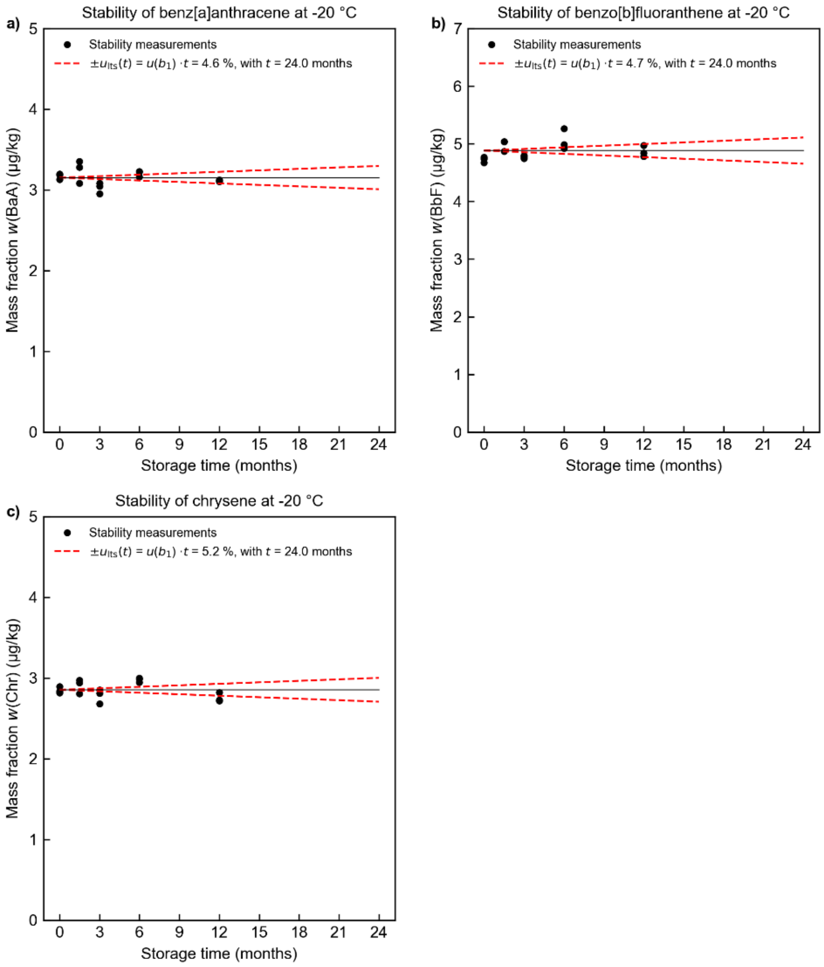

Fig. S4 Long-term stability of BaA, BbF and Chr at -20 °C with estimated relative standard uncertainty u_{lts}

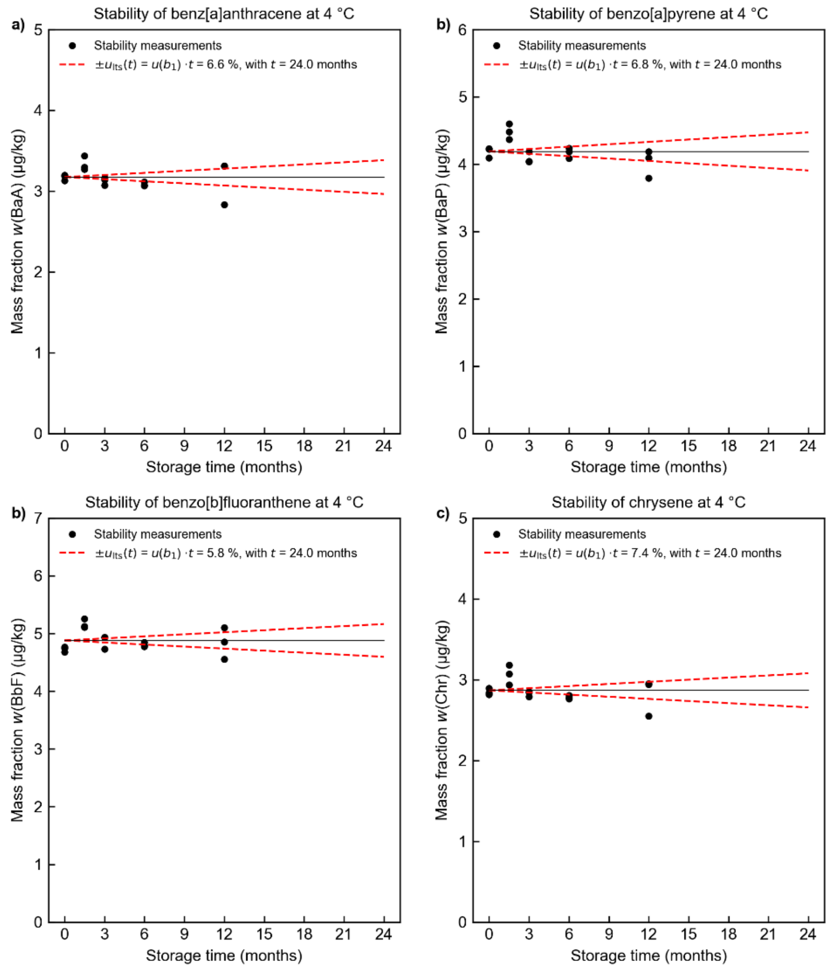

Fig. S5 Long-term stability of BaA, BaP, BbF and Chr at 4 °C with estimated relative standard uncertainty u_{lts}

Table S2 Results of the two-tailed t-test and analysis of the long-term stability at 4 °C for WP-CBR001

PAH	b_1 (µg · kg^{-1} · months^{-1})	b_0 (µg · kg^{-1})	$s(b_1)$ (µg · kg^{-1} · months^{-1})	t_{b1}	t_{crit}
BaA	-0.007703	3.208267	0.008786	0.877	2.160
BaP	-0.022024	4.288553	0.010603	2.077	2.160
BbF	-0.006438	4.909184	0.012144	0.530	2.160
Chr	-0.010466	2.915234	0.008651	1.210	2.160

PAH	s (b_1 = 0) (µg·kg^{-1}·months^{-1})	b_0 (b_1 = 0) = w_{lts}(PAH) (µg/kg)	$u_{lts}[w_{lts}$(PAH)] (µg/kg)	$u_{lts, r}[w_{lts}$(PAH)] (%)
BaA	0.008713	3.173606	0.209	6.589
BaP	0.011791	4.189447	0.283	6.755
BbF	0.011828	4.880214	0.284	5.817
Chr	0.008793	2.868138	0.211	7.358

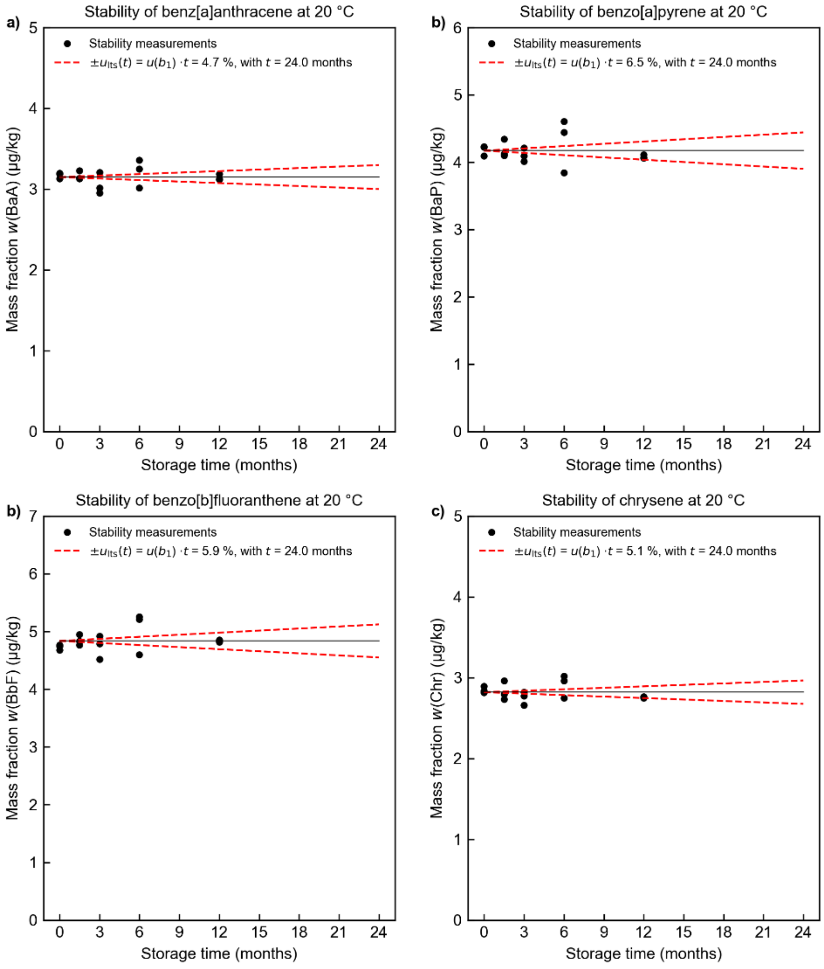

Fig. S6 Long-term stability of BaA, BaP, BbF and Chr at 20 °C with estimated relative standard uncertainty u_{lts}

Table S3 Results of the two-tailed t-test and analysis of the long-term stability at 20 °C for WP-CBR001

PAH	b_1 (µg · kg^{-1} · months^{-1})	b_0 (µg · kg^{-1})	$s(b_1)$ (µg · kg^{-1} · months^{-1})	t_{b1}	t_{crit}
BaA	0.000532	3.146771	0.006417	0.083	2.160
BaP	-0.005406	4.195899	0.011595	0.466	2.160
BbF	0.009652	4.793541	0.012092	0.798	2.160
Chr	-0.004076	2.838802	0.006168	0.661	2.160

PAH	s (b_1 = 0) (µg·kg^{-1}·months^{-1})	b_0 (b_1 = 0) = w_{lts}(PAH) (µg/kg)	$u_{lts}[w_{lts}$(PAH)] (µg/kg)	$u_{lts, r}[w_{lts}$(PAH)] (%)
BaA	0.006185	3.149166	0.148	4.714
BaP	0.011266	4.171573	0.270	6.482
BbF	0.011935	4.836977	0.286	5.922
Chr	0.006043	2.820462	0.145	5.142

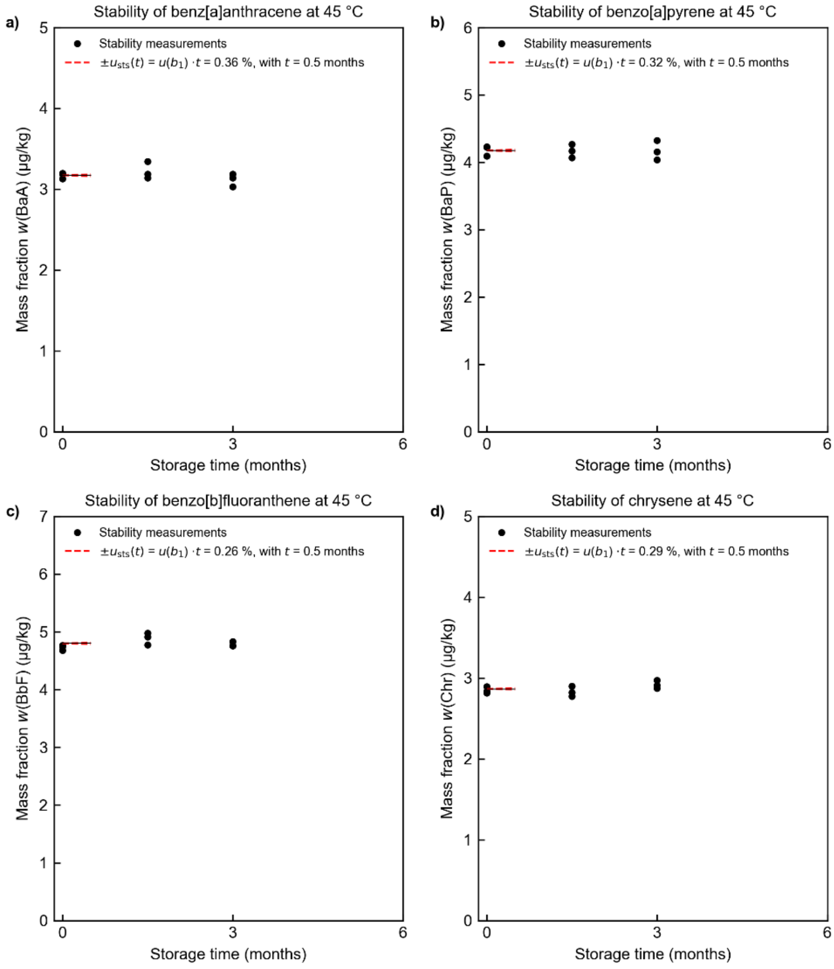

Fig. S7 Short-term stability of BaA, BbF and Chr at 45 °C with estimated relative standard uncertainty u_{sts}

5. Uncertainty estimation of the measurement steps

For each sample measurement, a standard uncertainty $u[w_{char,ij}(\text{PAH})]$ was calculated according to the following steps.

Definition of the output variable

Table S4 Definition of output variable (measurand)

Symbol	Quantity	Unit
$w_{char,ij}(\text{PAH})$	Mass fraction of native PAH (BaA, BaP, BbF or Chr) in the sample	µg/kg

Definition of the input variables

Table S5 Definition of input variables

Symbol	Quantity	Unit
$w_{ref}(\text{PAH})$	Certified mass fraction of the native PAH in the standard reference material SRM NIST 1647f	mg/kg
m_{ref}	Mass of standard reference material SRM NIST 1647f for preparation of reference stock solution	g
$m_{toluene,\ stock}$	Mass of toluene for the preparation of reference stock solution	g
$m_{stock,\ h}$	Mass of reference stock solution for preparation of intermediate reference solution z, *h* (*h* = 1 to 6)	g
$m_{toluene,\ z,\ h}$	Mass of toluene for the preparation of the intermediate reference solution z, *h* (*h* = 1 to 6)	g
$m_{z,\ h}$	Mass of the intermediate reference solution z, h for preparation of the calibration blends zy, *h* (*h* = 1 to 6)	g
$m_{yz,\ h}$	Mass of the spike solution y, h for preparation of the calibration blends yz, *h* (*h* = 1 to 6)	g
$m_{z,\ FBkF,\ h}$	Mass of the injection standard FBkF for the preparation of the calibration bends yz, *h* (*h* = 1 to 6)	g
m_{yx}	Mass of the spike solution y for the preparation of the sample blend yx	g
m_x	Mass of the sample for the preparation of the sample blend yx	g
R_{bx}	Measured isotope ratio (peak area ratio) of the quantifier ions of the native PAH and the deuterated DPAH in the sample blend yx	-
$R_{bz,h}$	Measured isotope ratio (peak area ratio) of the quantifier ions of the native PAH and the DPAH in the calibration blend yz, *h* (*h* = 1 to 6)	-

Definition of the model equation

The linear equation to describe the regression line of the calibration is given in Eq. (S1).

$$R_{\text{bz},h} = b_1 \cdot \frac{w_{\text{yz},h}(\text{PAH})}{w_{\text{yz},h}(\text{y})} + b_0 \qquad \text{(S1)}$$

$w_{yz,h}$(PAH):	Mass fraction of the native PAH in the calibration blend yz, *h* (*h* = 1 to 6) (ng/g)
$w_{yz,h}$(y):	Mass fraction of the spike solution y in the calibration blend yz, *h* (*h* = 1 to 6) (ng/g)
b_1:	Slope of the linear regression curve (g/ng)
b_0:	Axis intercept of the linear regression curve (-)

Linear regression analysis was performed with six calibration (reference) blends yz, *h* (*h* = 1 to 6) prepared from the intermediate reference solutions z, *h* (*h* = 1 to 6), the spike solution y and the injection standard FBkF. The mass fractions of the native PAHs in the individual calibration (reference) blends, $w_{yz,h}$(PAH), were calculated according to Eqs. S2 to S4.

$$w_{\mathrm{stock}}(\mathrm{PAH}) = w_{\mathrm{ref}}(\mathrm{PAH}) \cdot \frac{m_{\mathrm{ref}}}{m_{\mathrm{toluene,stock}} + m_{\mathrm{ref}}} \quad \text{(S2)}$$

$$w_{\mathrm{z},h}(\mathrm{PAH}) = w_{\mathrm{stock}}(\mathrm{PAH}) \cdot \frac{m_{\mathrm{stock},h}}{m_{\mathrm{toluene,z},h} + m_{\mathrm{stock},h}} \quad \text{(S3)}$$

$$w_{\mathrm{yz},h}(\mathrm{PAH}) = w_{\mathrm{z},h}(\mathrm{PAH}) \cdot \frac{m_{\mathrm{z},h}}{m_{\mathrm{z},h} + m_{\mathrm{yz},h} + m_{\mathrm{z,FBkF},h}} \quad \text{(S4)}$$

The mass fractions of the spike solution y in the individual calibration (reference) blends, $w_{yz,h}$(y), with *h* = 1 to 6, were calculated according to Eq. S5.

$$w_{\mathrm{yz},h}(\mathrm{y}) = \frac{m_{\mathrm{y},h}}{m_{\mathrm{yz},h} + m_{\mathrm{z},h} + m_{\mathrm{z,FBkF},h}} \quad \text{(S5)}$$

An ordinary least squares (OLS) fit model was used to determine the calibration parameters b_1 (slope) and b_0 (intercept).

The mass fraction of the native PAH (measurand) for a single sample measurement (*ij*) was calculated according to Eq. S6.

$$w_{\mathrm{char},ij}(\mathrm{PAH}) = \left(\frac{R_{\mathrm{bx},ij} - b_0}{b_1}\right) \cdot \frac{m_{\mathrm{yx},ij}}{m_{\mathrm{x},ij}} \quad \text{(S6)}$$

As explained in detail below, the uncertainty contribution of R_{bx} was omitted in the calculations because of a significant correlation to the uncertainty contribution from repeatability.

Uncertainty contribution from weighing process

For estimating the uncertainty contribution of the weighing processes, the approach described by Reichmuth et al. [1] was used. For each weighing, an air buoyancy correction was applied. Together with the uncertainty components of the balance, a combined standard uncertainty for each mass was calculated. Fig. S8 shows all input variables that were considered for the weighing process. Without air buoyancy correction, almost identical values for the mass fractions of the native PAHs were obtained, which means that the air buoyancy correction has only a

negligible influence. Moreover, the combined standard uncertainties for the masses were small contributors to the u_{char} uncertainty budget.

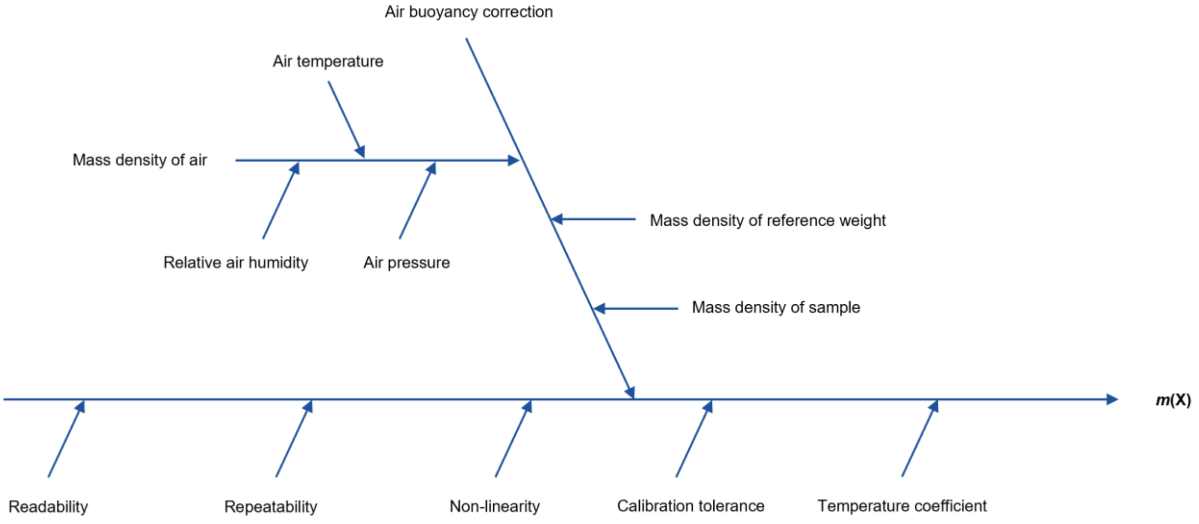

Fig. S8 Cause-and-effect diagram of weighing processes

Uncertainty estimation of the measurement steps

The uncertainty of the measurement steps, $u_{\mathrm{meas}}[w_{\mathrm{char}}(\mathrm{PAH})]$, was estimated by the mean value of the standard uncertainties of all measurements, $u_{\mathrm{meas}}[w_{\mathrm{char},ij}(\mathrm{PAH})]$, according to Eq. S7.

$$u_{\mathrm{meas}}[w_{\mathrm{char}}(\mathrm{PAH})] = \frac{1}{n} \cdot \frac{1}{k} \cdot \sum_{i=1}^{n} \sum_{j=1}^{k} u_{\mathrm{meas}}\left[w_{\mathrm{char},ij}(\mathrm{PAH})\right] \tag{S7}$$

Uncertainty budget of the measurement steps

Value	Std Unc	U95
4.052269	0.043207	0.086414

Description	Unc Component	Unc Percentage
m_ref	0.000275	0.004
m_stock_1	0.000152	0.001
m_stock_2	0.000149	0.001
m_stock_3	0.000137	0.001
m_stock_4	0.000127	0.001
m_stock_5	0.000174	0.002
m_stock_6	0.000156	0.001
m_toluene_stock	0.000168	0.002
m_toluene_z_1	0.000006	0.000
m_toluene_z_2	0.000011	0.000
m_toluene_z_3	0.000025	0.000
m_toluene_z_4	0.000036	0.000
m_toluene_z_5	0.000046	0.000
m_toluene_z_6	0.000055	0.000
m_x	0.001157	0.072
m_yx	0.001999	0.214
m_yz_1	0.000030	0.000
m_yz_2	0.000058	0.000
m_yz_3	0.000140	0.001
m_yz_4	0.000206	0.002
m_yz_5	0.000263	0.004
m_yz_6	0.000317	0.005
m_z_1	0.000025	0.000
m_z_2	0.000047	0.000
m_z_3	0.000113	0.001
m_z_4	0.000167	0.001
m_z_5	0.000211	0.002
m_z_6	0.000254	0.003
m_z_FBkF_1	0.000000	0.000
m_z_FBkF_2	0.000000	0.000
m_z_FBkF_3	0.000000	0.000
m_z_FBkF_4	0.000000	0.000
m_z_FBkF_5	0.000000	0.000
m_z_FBkF_6	0.000000	0.000
R_bz_1	0.003669	0.721
R_bz_2	0.005884	1.855
R_bz_3	0.005601	1.680
R_bz_4	0.008871	4.215
R_bz_5	0.012758	8.719
R_bz_6	0.021883	25.650
w_ref(BaP)	0.032575	56.839

Fig. S9 Print screen of the uncertainty budget of measurement j = 1 of bottle no. 197 (i = 4) of BaP calculated by METAS UncLib [2]

6. Uncertainty estimation of the repeatability

The uncertainty of the repeatability, $u_{rep}[w_{char}(\mathrm{PAH})]$, was estimated via the calculation of the standard deviation of the mean mass fraction within each bottle (i), s_{mean}, and calculation of the mean of all s_{mean} values according to Eqs. S8 to S10.

$$s_{\mathrm{mean}}\left[w_{\mathrm{char},i}(\mathrm{PAH})\right] = \sqrt{\frac{\sum_{j=1}^{k}\left(w_{\mathrm{char},ij}(\mathrm{PAH}) - w_{\mathrm{char},i}(\mathrm{PAH})\right)^2}{n-1}} \cdot \frac{1}{\sqrt{k}} \tag{S8}$$

$$w_{\mathrm{char},i} = \frac{1}{k} \cdot \sum_{j=1}^{k} w_{\mathrm{char},ij}(\mathrm{PAH}) \tag{S9}$$

$$u_{\mathrm{rep}}[w_{\mathrm{char}}(\mathrm{PAH})] = \frac{1}{n} \cdot \sum_{i=1}^{n} s_{\mathrm{mean}}\left[w_{\mathrm{char},i}(\mathrm{PAH})\right] \tag{S10}$$

7. Uncertainty estimation of the characterization of the material

Evaluation of possible correlations through the of cause-and-effect diagram

For the estimation of the standard uncertainty of the characterization process (u_{char}) the contributions of the measurement steps and the repeatability were assessed with the aid of the cause-and-effect diagram illustrated in Fig. S10. A major correlation obviously exists between the repeatability and the measured isotope ratio (peak area ratio) R_{bx}. While the precision of the GC-MS/MS measurement is included in both R_{bx} and the repeatability, the latter additionally takes into account uncertainties associated with the extraction step. Therefore, the uncertainty of R_{bx} was omitted in the calculation of the uncertainty of the individual measurement steps to avoid multiple counting. Other possible correlations were not considered significant and were therefore not taken into account. This procedure very likely leads to a slight overestimation of the standard uncertainty u_{char}.

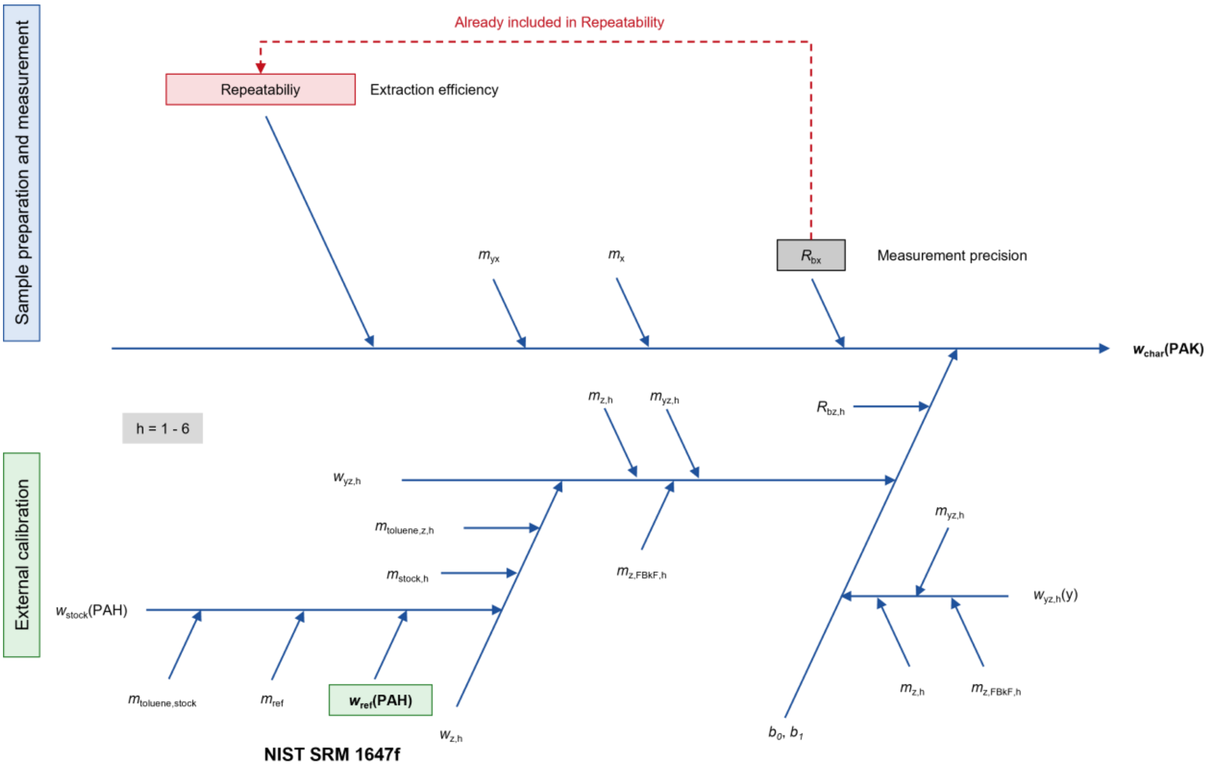

Fig. S10 Cause-and-effect diagram of analytical method for PAHs determination

Uncertainty estimation of the characterization of the material

In Tables S12 to S15 all raw data for calculating the uncertainty of the characterization of the material, $u_{char}[w_{char}(\text{PAH})]$, are presented. The uncertainty contributions from the measurement steps, $u_{meas}[w_{char}(\text{PAH})]$, and the repeatability, $u_{rep}[w_{char}(\text{PAH})]$, were combined to $u_{char}[w_{char}(\text{PAH})]$ according to Eq. S11.

$$u_{char}[w_{char}(\text{PAH})] = \sqrt{u_{meas}^2[w_{char}(\text{PAH})] + u_{rep}^2[w_{char}(\text{PAH})]} \quad \text{(S11)}$$

Table S6 Uncertainty contributions of the measurement and repeatability parts to $u_{char}[w_{char}(BaA)]$. * Percentage contribution of $u_i^2[w_{char}(BaA)]$ to $u_{char}^2[w_{char}(BaA)]$

**BaA**	Contribution *i*	
	Measurement (meas)	Repeatability (rep)
w_{char}(BaA) (µg/kg)	3.1724	
$u_i[w_{char}$(BaA) (µg/kg)	0.0270	0.0463
$u_{i,r}[w_{char}$(BaA)] (-)	0.0085	0.0146
%$u_{char}[w_{char}$(BaA)]* (%)	25.3	74.7
$u_{char}[w_{char}$(BaA)] (µg/kg)	0.0536	

Table S7 Uncertainty contributions of the measurement and repeatability parts to $u_{char}[w_{char}(BaP)]$. * Percentage contribution of $u_i^2[w_{char}(BaP)]$ to $u_{char}^2[w_{char}(BaP)]$

**BaP**	Contribution *i*	
	Measurement (meas)	Repeatability (rep)
w_{char}(BaP) (µg/kg)	4.1826	
$u_i[w_{char}$(BaP) (µg/kg)	0.0450	0.0982
$u_{i,r}[w_{char}$(BaP)] (-)	0.0108	0.0235
%$u_{char}[w_{char}$(BaP)]* (%)	17.4	82.6
$u_{char}[w_{char}$(BaP)] (µg/kg)	0.1080	

Table S8 Uncertainty contributions of the measurement and repeatability parts to $u_{char}[w_{char}(BbF)]$. * Percentage contribution of $u_i^2[w_{char}(BbF)]$ to $u_{char}^2[w_{char}(BbF)]$

**BbF**	Contribution *i*	
	Measurement (meas)	Repeatability (rep)
w_{char}(BbF) (µg/kg)	4.7291	
$u_i[w_{char}$(BbF) (µg/kg)	0.0426	0.0830
$u_{i,r}[w_{char}$(BbF)] (-)	0.0090	0.0175
%$u_{char}[w_{char}$(BbF)]* (%)	20.9	79.1
$u_{char}[w_{char}$(BbF)] (µg/kg)	0.0933	

Table S9 Uncertainty contributions of the measurement and repeatability parts to $u_{char}[w_{char}(Chr)]$. * Percentage contribution of $u_i^2[w_{char}(Chr)]$ to $u_{char}^2[w_{char}(Chr)]$

**Chr**	Contribution *i*	
	Measurement (meas)	Repeatability (rep)
w_{char}(Chr) (µg/kg)	2.8471	
$u_i[w_{char}$(Chr) (µg/kg)	0.0282	0.0498
$u_{i,r}[w_{char}$(Chr)] (-)	0.00991	0.01748
%$u_{char}[w_{char}$(Chr)]* (%)	24.3	75.7
$u_{char}[w_{char}$(Chr)] (µg/kg)	0.0572	

8. Uncertainty budget

Table S10 Mass fractions and estimated combined and expanded uncertainties of BaA in WP-CBR001. * Contribution to combined standard uncertainty. ** Percentage contribution of $u_i^2[w_{char}(BaA)]$ to $u_c^2[w_{char}(BaA)]$

BaA	Contribution i			
	Characterization (char)	Homogeneity (hom)	Long-term stability (lts)	Short-term stability (sts)
w_i(BaA) (µg/kg)	3.1724	3.1724	3.1524	3.1717
$u_i[w_i$(BaA) (µg/kg)	0.0536	0.0277	0.1444	0.0114
$u_{i,r}[w_i$(BaA)] (-)	0.0169	0.0087	0.0458	0.0036
$u_i[w_{char}$(BaA)]* (µg/kg)	0.0536	0.0277	0.1453	0.0113
%$u_c[w_{char}$(BaA)]** (%)	11.5	3.1	84.9	0.5
$u_c[w_{char}$(BaA)] (µg/kg)	0.1577			
$U[w_{char}$(BaA)] (µg/kg)	0.32			

Table S11 Mass fractions and estimated combined and expanded uncertainties of BbF in WP-CBR001. * Contribution to combined standard uncertainty. ** Percentage contribution of $u_i^2[w_{char}(BbF)]$ to $u_c^2[w_{char}(BbF)]$

BbF	Contribution i			
	Characterization (char)	Homogeneity (hom)	Long-term stability (lts)	Short-term stability (sts)
w_i(BbF) (µg/kg)	4.7291	4.7291	4.8821	4.8005
$u_i[w_i$(BbF) (µg/kg)	0.0933	0.0497	0.2269	0.0126
$u_{i,r}[w_i$(BbF)] (-)	0.0197	0.0105	0.0465	0.0026
$u_i[w_{char}$(BbF)]* (µg/kg)	0.0933	0.0497	0.2198	0.0124
%$u_c[w_{char}$(BbF)]** (%)	14.6	4.1	81.0	0.3
$u_c[w_{char}$(BbF)] (µg/kg)	0.2442			
$U[w_{char}$(BbF)] (µg/kg)	0.49			

Table S12 Mass fractions and estimated combined and expanded uncertainties of Chr in WP-CBR001. * Contribution to combined standard uncertainty. ** Percentage contribution of $u_i^2[w_{char}(Chr)]$ to $u_c^2[w_{char}(Chr)]$

Chr	Contribution i			
	Characterization (char)	Homogeneity (hom)	Long-term stability (lts)	Short-term stability (sts)
w_i(Chr) (µg/kg)	2.8471	2.8471	2.8541	2.8661
$u_i[w_i$(Chr) (µg/kg)	0.0572	0.0300	0.1477	0.0083
$u_{i,r}[w_i$(Chr)] (-)	0.0201	0.0105	0.0517	0.0029
$u_i[w_{char}$(Chr)]* (µg/kg)	0.0572	0.0300	0.1473	0.0082
%$u_c[w_{char}$(Chr)]** (%)	12.6	3.5	83.7	0.3
$u_c[w_{char}$(Chr)] (µg/kg)	0.1610			
$U[w_{char}$(Chr)] (µg/kg)	0.33			

9. Gravimetric mass fractions

$$w_{\text{stock sol}}(\text{PAH}) = \frac{m_{\text{SM}}(\text{PAH}) \cdot w_{\text{SM}}(\text{PAH})}{m_{\text{bulk stock sol}}} \cdot hom_{\text{stock sol}} \qquad \text{(S12)}$$

$w_{\text{stock sol}}$(PAH)	Mass fraction of PAH of stock solution
m_{SM}(PAH)	Mass of PAH standard material (CRM)
w_{SM}(PAH)	Mass fraction of PAH of standard material (CRM)
$m_{\text{bulk stock sol}}$	Mass of final stock solution in acetonitrile
$hom_{\text{stock sol}}$	Homogenizing factor

Table S13 Input values used to calculate the mass fractions of the PAHs in the stock solution

PAH	m_{SM}(PAH) (g)	$u_{\text{r}}[m_{\text{SM}}(\text{PAH})]$ (%)	w_{SM}(PAH) (mg/kg)	$u_{\text{r}}[w_{\text{SM}}(\text{PAH})]$ (%)	$m_{\text{bulk stock sol}}$ (g)	$u_{\text{r}}[m_{\text{bulk stock sol}}]$ (%)	$hom_{\text{stock sol}}$ (-)	$u_{\text{r}}[hom_{\text{stock sol}}]$ (%)
BaA	0.02099	0.65	980'000	0.15	197.94195	0.01	1	0.03
BaP	0.02780	0.65	992'000	0.20				
BbF	0.03337	0.65	942'000	1.25				
Chr	0.01896	0.65	976'000	0.25				

$$w_{\text{spike sol}}(\text{PAH}) = \frac{m_{\text{stock sol}} \cdot w_{\text{stock sol}}(\text{PAH})}{m_{\text{bulk spike sol}}} \cdot hom_{\text{spike sol}} \qquad \text{(S13)}$$

$w_{\text{spike sol}}$(PAH)	Mass fraction of PAH of spike solution
$m_{\text{stock sol}}$	Mass of stock solution
$m_{\text{bulk spike sol}}$	Mass of final spike solution in acetonitrile
$hom_{\text{spike sol}}$	Homogenizing factor

Table S14 Input values used to calculate the mass fractions of the PAHs in the spike solution

PAH	$m_{\text{stock sol}}$(PAH) (g)	$u_{\text{r}}[\text{m}_{\text{stock sol}}(\text{PAH})]$ (%)	$m_{\text{bulk spike sol}}$ (g)	$u_{\text{r}}[\text{m}_{\text{bulk spike sol}}]$ (%)	$hom_{\text{spike sol}}$ (-)	$u_{\text{r}}[hom_{\text{spike sol}}]$ (%)
BaA	1.43176	0.01	94.15864	0.01	1	0.03
BaP						
BbF						
Chr						

$$w_{\text{whey cont}}(\text{PAH}) = \frac{m_{\text{spike sol}} \cdot w_{\text{spike sol}}(\text{PAH})}{m_{\text{bulk whey cont}}} \cdot hom_{\text{whey cont}} \tag{S14}$$

$w_{\text{whey cont}}(\text{PAH})$	Mass fraction of PAH of contaminated whey
$m_{\text{spike sol}}$	Mass of spike solution
$m_{\text{bulk whey cont}}$	Mass of final contaminated whey
$hom_{\text{whey cont}}$	Homogenizing factor

Table S15 Input values used to calculate the mass fractions of the PAHs in the contaminated whey

PAH	$m_{\text{spike sol}}(\text{PAH})$ (g)	$u_r[m_{\text{spike sol}}(\text{PAH})]$ (%)	$m_{\text{bulk whey cont}}$ (g)	$u_r[\text{m}_{\text{bulk whey cont}}]$ (g)	$hom_{\text{whey cont}}$ (-)	$u_r[hom_{\text{whey cont}}]$ (%)
BaA	59.11954	0.01	90088	250	1	0.03
BaP						
BbF						
Chr						

$$w_{\text{grav}}(\text{PAH}) = w_{\text{whey cont}}(\text{PAH}) \cdot \frac{100 - w_{\text{water}}}{w_{\text{dry mass}}} \tag{S15}$$

$w_{\text{grav}}(\text{PAH})$	Gravimetric mass fraction of PAH of final whey protein powder
w_{water}	Value of experience of the water content of the whey protein powder
$w_{\text{dry mass}}$	Dry mass of contaminated whey

Table S16 Input values used to calculate the mass fractions of the PAHs in the final whey protein powder

PAH	w_{water} (g/100 g)	$u_r[w_{\text{water}}]$ (g/100 g)	$w_{\text{dry mass}}$ (g/100 g)	$u_r[w_{\text{dry mass}}]$ (g/100 g)
BaA	5.0	0.5	30.5	0.1
BaP				
BbF				
Chr				

10. Interlaboratory comparison study

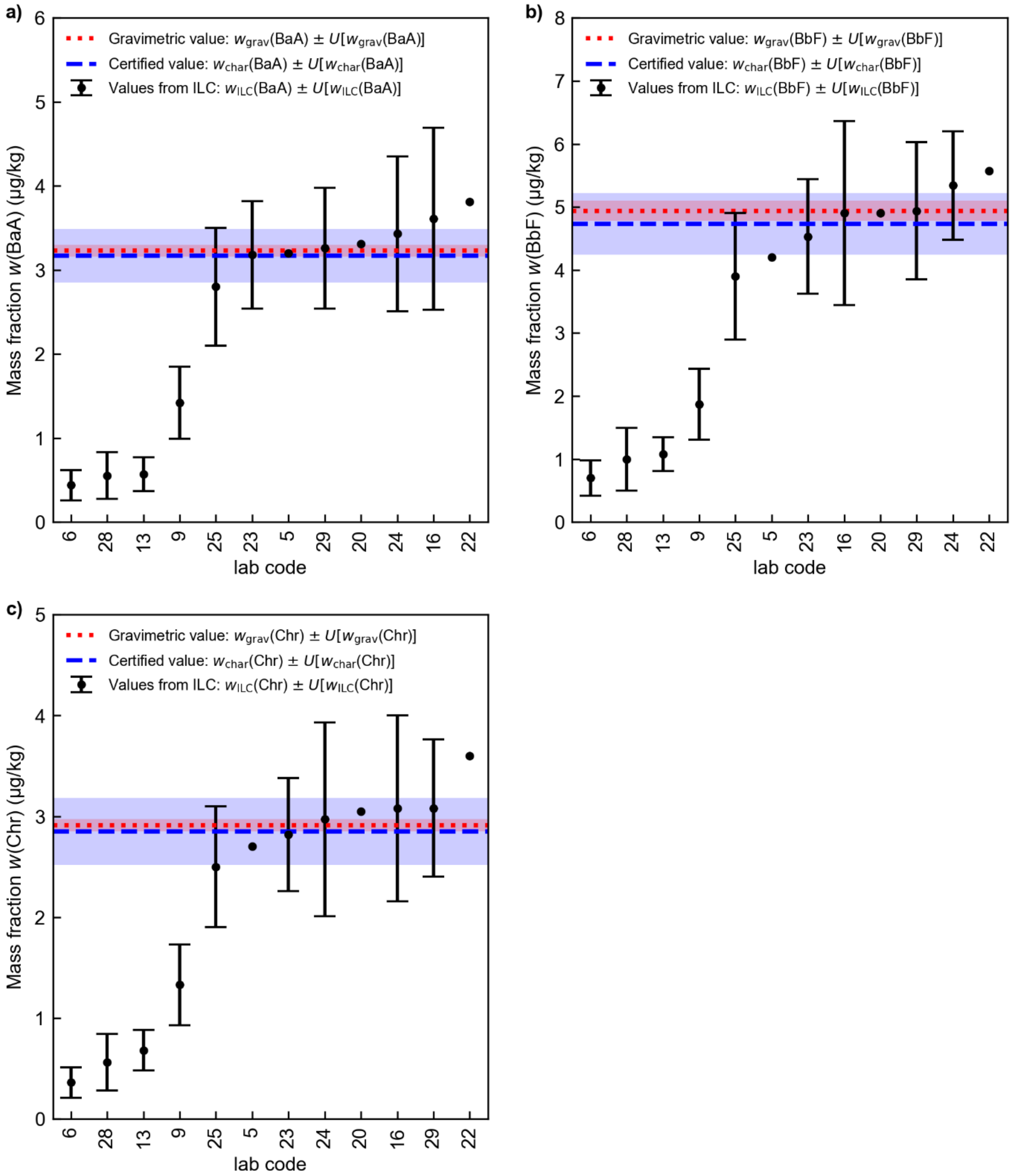

Fig. S11 Comparison of the certified mass fractions of BaA, BbF and Chr with the results of the interlaboratory comparison study. Note: the results of the interlaboratory comparison are given on a dry mass basis whereas the certified value is based on the material as is. For data points without uncertainty bars no uncertainty values were submitted by the corresponding laboratories

11. Solvent extraction efficiencies

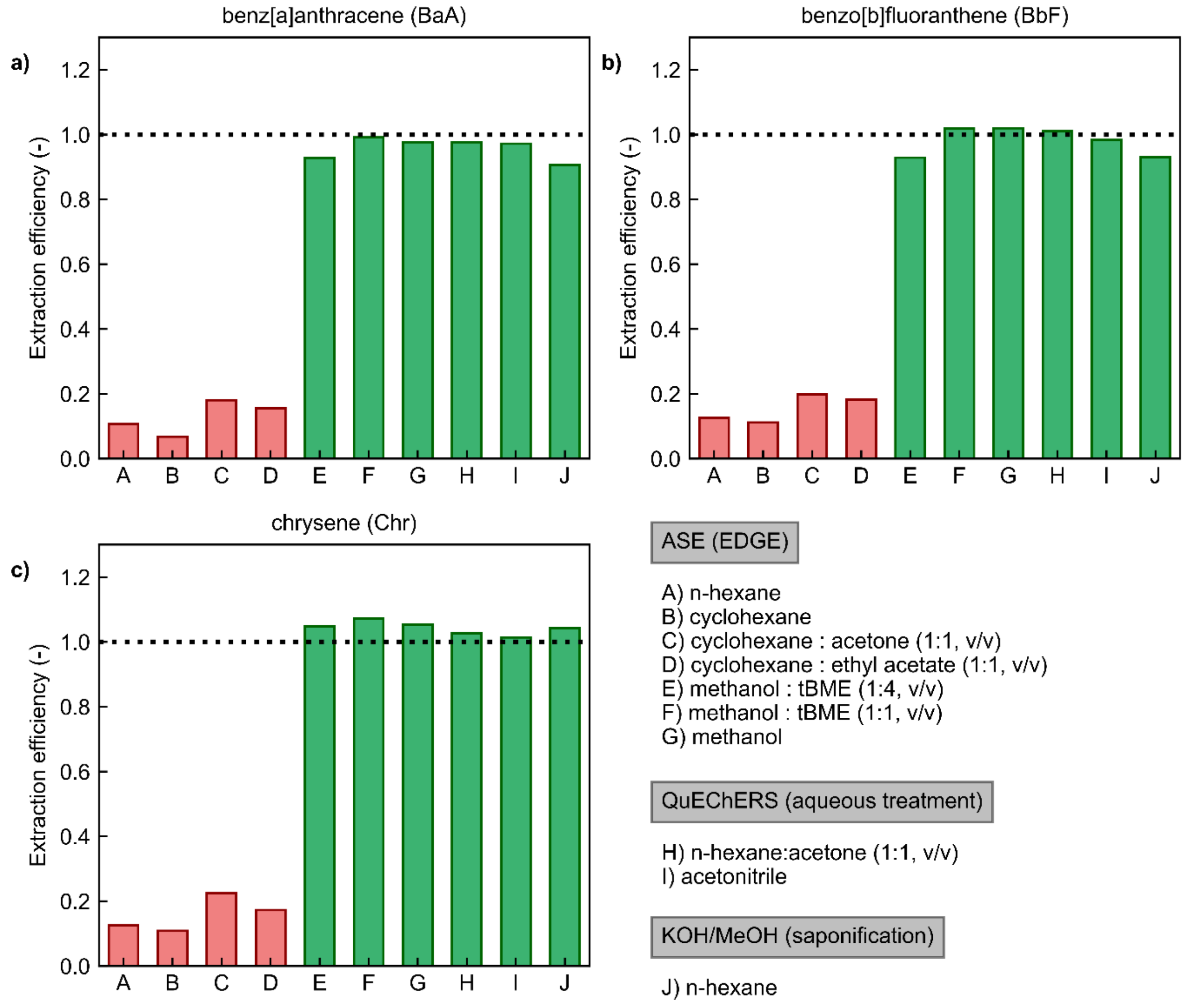

Fig. S12 Solvent extraction efficiencies of BaA, BbF and Chr for WP-CBR001

12. Raw data for homogeneity assessment, characterization and value assignment

Table S17 Measured mass fractions of BaA (homogeneity study) and calculation of w_{char}(BaA)* and $u_{char}[w_{char}$(BaA)]

**BaA**		Measurement j								
		1		2		3				
Bottle	i	$w_{hom,ij}$(BaA)	$u[w_{hom,ij}$(BaA)]	$w_{hom,ij}$(BaA)	$u[w_{hom,ij}$(BaA)]	$w_{hom,ij}$(BaA)	$u[w_{hom,ij}$(BaA)]	$w_{char,i}$(BaA)	$u_{meas}[w_{char,i}$(BaA)]	$s_{mean}[w_{char,i}$(BaA)]
9	1	3.1133	0.0266	3.2337	0.0275	3.1679	0.0269	3.1716	0.0270	0.0348
75	2	3.1177	0.0265	3.1510	0.0269	3.1028	0.0265	3.1239	0.0266	0.0142
61	3	3.1022	0.0264	3.0492	0.0260	3.1701	0.0269	3.1072	0.0264	0.0350
197	4	3.0853	0.0263	3.1936	0.0271	3.1542	0.0268	3.1444	0.0267	0.0317
134	5	3.1006	0.0264	3.0802	0.0263	3.2718	0.0277	3.1509	0.0268	0.0608
317	6	3.2221	0.0273	3.4056	0.0288	3.1984	0.0272	3.2753	0.0278	0.0655
255	7	3.1481	0.0269	3.0731	0.0275	3.2516	0.0262	3.1576	0.0268	0.0518
500	8	3.1189	0.0266	3.3243	0.0281	3.2737	0.0277	3.2389	0.0275	0.0618
578	9	3.2253	0.0274	3.0836	0.0263	3.1637	0.0269	3.1575	0.0269	0.0410
620	10	3.0659	0.0261	3.2805	0.0278	3.2441	0.0274	3.1968	0.0271	0.0663
**w_{char}(BaA) (µg/kg)***								**3.1724**		
$u_{meas}[w_{char}$(BaA)] (µg/kg)									0.0270	
$u_{rep}[w_{char}$(BaA)] (µg/kg)										0.0463
**$u_{char}[w_{char}$(BaA)] (µg/kg)**									**0.0536**	
**$u_{char,r}[w_{char}$(BaA)] (-)**									**0.0169**	
**$u_{char,r}[w_{char}$(BaA)] (%)**									**1.69**	

* w_{hom}(BaA) = w_{char}(BaA)

Table S18 Measured mass fractions of BaP (homogeneity study) and calculation of w_{char}(BaP)* and $u_{char}[w_{char}$(BaP)]

**BaP**		Measurement j								
		1		2		3				
Bottle	i	$w_{hom,ij}$(BaP)	$u[w_{hom,ij}$(BaP)]	$w_{hom,ij}$(BaP)	$u[w_{hom,ij}$(BaP)]	$w_{hom,ij}$(BaP)	$u[w_{hom,ij}$(BaP)]	$w_{char,i}$(BaP)	$u_{meas}[w_{char,i}$(BaP)]	$s_{mean}[w_{char,i}$(BaP)]
9	1	3.9889	0.0421	4.4878	0.0490	4.1625	0.0449	4.2130	0.0453	0.1462
75	2	3.9523	0.0418	4.2828	0.0462	4.1181	0.0437	4.1177	0.0439	0.0954
61	3	4.0208	0.0427	4.1604	0.0450	4.3238	0.0473	4.1683	0.0450	0.0875
197	4	4.0523	0.0432	4.3419	0.0476	4.1415	0.0445	4.1786	0.0451	0.0856
134	5	4.0917	0.0439	3.9977	0.0424	4.5063	0.0498	4.1986	0.0454	0.1563
317	6	4.2010	0.0454	4.2860	0.0462	4.1187	0.0438	4.2019	0.0451	0.0483
255	7	4.1292	0.0439	4.0193	0.0428	4.3886	0.0485	4.1790	0.0450	0.1095
500	8	4.0041	0.0424	4.3632	0.0475	4.2983	0.0469	4.2219	0.0456	0.1105
578	9	4.3164	0.0465	3.9818	0.0421	4.0500	0.0430	4.1161	0.0439	0.1021
620	10	4.1620	0.0452	4.3013	0.0464	4.2304	0.0459	4.2312	0.0458	0.0402
**w_{char}(BaP) (µg/kg)***								**4.1826**		
$u_{meas}[w_{char}$(BaP)] (µg/kg)									0.0450	
$u_{rep}[w_{char}$(BaP)] (µg/kg)										0.0982
**$u_{char}[w_{char}$(BaP)] (µg/kg)**									**0.1080**	
**$u_{char,r}[w_{char}$(BaP)] (-)**									**0.258**	
**$u_{char,r}[w_{char}$(BaP)] (%)**									**2.58**	

* w_{hom}(BaP) = w_{char}(BaP)

Table S19 Measured mass fractions of BbF (homogeneity study) and calculation of w_{char}(BbF)* and $u_{char}[w_{char}$(BbF)]

**BbF**		Measurement *j*								
		1		2		3				
Bottle	*i*	$w_{hom,ij}$(BbF)	$u[w_{hom,ij}$(BbF)]	$w_{hom,ij}$(BbF)	$u[w_{hom,ij}$(BbF)]	$w_{hom,ij}$(BbF)	$u[w_{hom,ij}$(BbF)]	$w_{char,i}$(BbF)	$u_{meas}[w_{char,i}$(BbF)]	$s_{mean}[w_{char,i}$(BbF)]
9	1	4.5139	0.0398	4.8180	0.0434	4.7807	0.0434	4.7042	0.0422	0.0957
75	2	4.5709	0.0407	4.7935	0.0431	4.7419	0.0424	4.7021	0.0421	0.0673
61	3	4.4224	0.0388	4.6467	0.0419	4.8958	0.0450	4.6550	0.0419	0.1367
197	4	4.5993	0.0411	4.7046	0.0426	4.7963	0.0435	4.7000	0.0424	0.0569
134	5	4.7412	0.0430	4.6662	0.0419	4.9287	0.0451	4.7787	0.0433	0.0780
317	6	4.7816	0.0434	4.7364	0.0424	4.7147	0.0421	4.7442	0.0427	0.0197
255	7	4.8786	0.0440	4.6257	0.0415	4.8661	0.0448	4.7901	0.0434	0.0823
500	8	4.5827	0.0407	4.9350	0.0450	4.7107	0.0426	4.7428	0.0428	0.1030
578	9	4.9359	0.0448	4.5880	0.0408	4.6129	0.0410	4.7123	0.0422	0.1121
620	10	4.7413	0.0433	4.9045	0.0444	4.6373	0.0417	4.7610	0.0431	0.0778
**w_{char}(BbF) (μg/kg)***								**4.7291**		
$u_{meas}[w_{char}$(BbF)] (μg/kg)									0.0426	
$u_{rep}[w_{char}$(BbF)] (μg/kg)										0.0830
**$u_{char}[w_{char}$(BbF)] (μg/kg)**									**0.0933**	
**$u_{char,r}[w_{char}$(BbF)] (-)**									**0.0197**	
**$u_{char,r}[w_{char}$(BbF)] (%)**									**1.97**	

* w_{hom}(BbF) = w_{char}(BbF)

Table S20 Measured mass fractions of Chr (homogeneity study) and calculation of w_{char}(Chr)* and $u_{char}[w_{char}(Chr)]$

**Chr**		Measurement *j*								
		1		2		3				
Bottle	*i*	$w_{hom,ij}$(Chr)	$u[w_{hom,ij}(Chr)]$	$w_{hom,ij}$(Chr)	$u[w_{hom,ij}(Chr)]$	$w_{hom,ij}$(Chr)	$u[w_{hom,ij}(Chr)]$	$w_{char,i}$(Chr)	$u_{meas}[w_{char,i}(Chr)]$	$s_{mean}[w_{char,i}(Chr)]$
9	1	2.7529	0.0268	2.8534	0.0280	2.9693	0.0301	2.8585	0.0283	0.0625
75	2	2.8439	0.0282	2.9179	0.0290	2.6364	0.0252	2.7994	0.0274	0.0843
61	3	2.7903	0.0274	2.6764	0.0261	2.7729	0.0274	2.7466	0.0270	0.0354
197	4	2.8082	0.0277	2.7538	0.0271	2.8383	0.0281	2.8001	0.0276	0.0247
134	5	2.7697	0.0273	2.7660	0.0271	3.0024	0.0305	2.8461	0.0283	0.0782
317	6	2.8802	0.0288	3.0133	0.0303	2.9374	0.0292	2.9436	0.0294	0.0386
255	7	2.8048	0.0273	2.8069	0.0277	2.9657	0.0300	2.8591	0.0284	0.0533
500	8	2.7811	0.0272	2.9196	0.0291	2.9522	0.0299	2.8843	0.0287	0.0525
578	9	2.8690	0.0282	2.7794	0.0272	2.9025	0.0289	2.8503	0.0281	0.0368
620	10	2.8417	0.0285	2.8624	0.0282	2.9448	0.0298	2.8830	0.0288	0.0315
**w_{char}(Chr) (µg/kg)***								**2.8471**		
$u_{meas}[w_{char}(Chr)]$ (µg/kg)									0.0282	
$u_{rep}[w_{char}(Chr)]$ (µg/kg)										0.0498
**$u_{char}[w_{char}(Chr)]$ (µg/kg)**									**0.0572**	
**$u_{char,r}[w_{char}(Chr)]$ (-)**									**0.201**	
**$u_{char,r}[w_{char}(Chr)]$ (%)**									**2.01**	

* w_{hom}(Chr) = w_{char}(Chr)

13. Raw data for stability assessment

Table S21 Measured mass fractions of BaA for stability assessment. * For the t = 0 time point, data from the homogeneity study were used: $w_{\mathrm{lts\ or\ sts},ij}(BaA) = \frac{1}{n}\sum_{i=1}^{n} w_{\mathrm{hom},ij}(BaA)$

**BaA**		-20 °C				+4 °C				RT (+20 °C)				+45 °C			
		Bottle	Measurement *j*			Bottle	Measurement *j*			Bottle	Measurement *j*			Bottle	Measurement *j*		
Storage time (months)	*i*		1	2	3		1	2	3		1	2	3		1	2	3
			$w_{\mathrm{lts},ij}$(BaA) (µg/kg)				$w_{\mathrm{lts},ij}$(BaA) (µg/kg)				$w_{\mathrm{lts},ij}$(BaA) (µg/kg)				$w_{\mathrm{sts},ij}$(BaA) (µg/kg)		
0	1	Hom*	3.1299	3.1875	3.1998	Hom*	3.1299	3.1875	3.1998	Hom*	3.1299	3.1875	3.1998	Hom*	3.1299	3.1875	3.1998
1.5	2	103	3.0836	3.3556	3.2819	645	3.4350	3.2961	3.2683	145	3.1274	3.2269	3.1340	586	3.3442	3.1399	3.1889
3	3	12	3.0477	3.0834	2.9548	312	3.1647	3.0743	3.1395	168	2.9511	3.2059	3.0173	249	3.1862	3.0308	3.1385
6	4	108	3.1604	3.2263	3.2258	198	3.0711	3.0652	3.1143	31	3.3569	3.0166	3.2489	-----	-----	-----	-----
12	5	298	3.1257	3.1013	3.1217	368	3.3137	3.3120	2.8327	45	3.1197	3.1890	3.1266	-----	-----	-----	-----

Table S22 Measured mass fractions of BaP for stability assessment. * For the t = 0 time point, data from the homogeneity study were used: $w_{\mathrm{lts\ or\ sts},ij}(BaP) = \frac{1}{n}\sum_{i=1}^{n} w_{\mathrm{hom},ij}(BaP)$

**BaP**		-20 °C				+4 °C				RT (+20 °C)				+45 °C			
		Bottle	Measurement *j*			Bottle	Measurement *j*			Bottle	Measurement *j*			Bottle	Measurement *j*		
Storage time (months)	*i*		1	2	3		1	2	3		1	2	3		1	2	3
			$w_{\mathrm{lts},ij}$(BaP) (µg/kg)				$w_{\mathrm{lts},ij}$(BaP) (µg/kg)				$w_{\mathrm{lts},ij}$(BaP) (µg/kg)				$w_{\mathrm{sts},ij}$(BaP) (µg/kg)		
0	1	Hom*	4.0919	4.2222	4.2338	Hom*	4.0919	4.2222	4.2338	Hom*	4.0919	4.2222	4.2338	Hom*	4.0919	4.2222	4.2338
1.5	2	103	4.1717	4.2044	4.5226	645	4.4802	4.3715	4.6015	145	4.0975	4.1254	4.3437	586	4.1713	4.0655	4.2702
3	3	12	4.1373	4.2390	4.0461	312	4.0341	4.0429	4.1855	168	4.0133	4.2139	4.0909	249	4.1532	4.0396	4.3242
6	4	108	4.3932	4.2760	4.4500	198	4.1885	4.0874	4.2347	31	4.6052	3.8441	4.4463	-----	-----	-----	-----
12	5	298	4.1781	4.1033	4.0535	368	4.0913	4.1840	3.7922	45	4.0706	4.1125	4.0622	-----	-----	-----	-----

Table S23 Measured mass fractions of BbF for stability assessment. * For the t = 0 time point, data from the homogeneity study were used: $w_{\text{lts or sts},ij}(BbF) = \frac{1}{n}\sum_{i=1}^{n} w_{\text{hom},ij}(BbF)$

**BbF**		-20 °C				+4 °C				RT (+20 °C)				+45 °C			
		Bottle	Measurement j			Bottle	Measurement j			Bottle	Measurement j			Bottle	Measurement j		
Storage time (months)	i		1	2	3		1	2	3		1	2	3		1	2	3
			$w_{\text{lts},ij}$(BbF) (µg/kg)				$w_{\text{lts},ij}$(BbF) (µg/kg)				$w_{\text{lts},ij}$(BbF) (µg/kg)				$w_{\text{sts},ij}$(BbF) (µg/kg)		
0	1	Hom*	4.6768	4.7419	4.7685	Hom*	4.6768	4.7419	4.7685	Hom*	4.6768	4.7419	4.7685	Hom*	4.6768	4.7419	4.7685
1.5	2	103	4.8720	5.0387	5.0383	645	5.1250	5.1103	5.2557	145	4.9532	4.7657	4.8311	586	4.9110	4.7767	4.9767
3	3	12	4.7480	4.7746	4.8010	312	4.9329	4.7285	4.9315	168	4.5224	4.9217	4.7909	249	4.7569	4.7612	4.8346
6	4	108	4.9237	4.9895	5.2622	198	4.7739	4.7997	4.8472	31	5.2154	4.6017	5.2528	-----	-----	-----	-----
12	5	298	4.9736	4.7809	4.8412	368	4.8536	5.1016	4.5561	45	4.8560	4.8373	4.8195	-----	-----	-----	-----

Table S24 Measured mass fractions of Chr for stability assessment. * For the t = 0 time point, data from the homogeneity study were used: $w_{\text{lts or sts},ij}(Chr) = \frac{1}{n}\sum_{i=1}^{n} w_{\text{hom},ij}(Chr)$

**Chr**		-20 °C				+4 °C				RT (+20 °C)				+45 °C			
		Bottle	Measurement j			Bottle	Measurement j			Bottle	Measurement j			Bottle	Measurement j		
Storage time (months)	i		1	2	3		1	2	3		1	2	3		1	2	3
			$w_{\text{lts},ij}$(Chr) (µg/kg)				$w_{\text{lts},ij}$(Chr) (µg/kg)				$w_{\text{lts},ij}$(Chr) (µg/kg)				$w_{\text{sts},ij}$(Chr) (µg/kg)		
0	1	Hom*	2.8142	2.8349	2.8922	Hom*	2.8142	2.8349	2.8922	Hom*	2.8142	2.8349	2.8922	Hom*	2.8142	2.8349	2.8922
1.5	2	103	2.8056	2.9393	2.9748	645	3.1830	3.0723	2.9389	145	2.7350	2.9633	2.7977	586	2.8983	2.7740	2.8216
3	3	12	2.8112	2.8517	2.6832	312	2.7880	2.8519	2.8421	168	2.6626	2.7737	2.8200	249	2.9089	2.8763	2.9742
6	4	108	2.9878	3.0011	2.9485	198	2.7806	2.8059	2.7665	31	3.0213	2.7468	2.9639	-----	-----	-----	-----
12	5	298	2.7277	2.7156	2.8240	368	2.9441	2.9545	2.5529	45	2.7505	2.7638	2.7670	-----	-----	-----	-----

References

1. Reichmuth A, Wunderli S, Weber M, Meyer VR. The uncertainty of weighing data obtained with electronic analytical balances. Microchim Acta 2004;148:133-141. https://doi.org/10.1007/s00604-004-0278-3

2. Zeier M, Hoffmann J, Wollensack M. Metas.UncLib - a measurement uncertainty calculator for advanced problems. Metrologia 2012;49:809-15. https://doi.org/10.1088/0026-1394/49/6/809
